# Comparative Efficacy and Safety of Swallowed Topical Corticosteroids in Eosinophilic Esophagitis: A Network Meta-Analysis

**DOI:** 10.3390/jcm14217823

**Published:** 2025-11-04

**Authors:** Alfredo J. Lucendo, Ángel Arias, Celia Álvarez-Bueno, Vicente Martínez-Vizcaino, Iván Redondo-Cavero

**Affiliations:** 1 Department of Gastroenterology, Hospital General de Tomelloso, 13700 Tomelloso, Spain; 2 Centro de Investigación Biomédica en Red Enfermedades Hepáticas y Digestivas (CIBEREHD), 28029 Madrid, Spain; aariasa@sescam.jccm.es; 3 Instituto de Investigación Sanitaria de Castilla-La Mancha (IDISCAM), 45004 Toledo, Spain; 4 Instituto de Investigación Sanitaria Princesa, 28006 Madrid, Spain; 5 Research Unit, Hospital Universitario La Mancha Centro, 13600 Alcázar de San Juan, Spain; 6 Health Science Faculty-HM Hospitals, Camilo José Cela University, 28036 Madrid, Spain; 7 Health and Social Research Center, Age-ABC Research Group, University of Castilla-La Mancha, 16002 Cuenca, Spain; celia.alvarezbueno@uclm.es (C.Á.-B.); vicente.martinez@uclm.es (V.M.-V.); 8 Facultad de Ciencias de la Salud, Universidad Autónoma de Chile, Talca 3460000, Chile; 9 CarVasCare Research Group, Faculty of Nursing, University of Castilla-La Mancha, 16002 Cuenca, Spain; ivan.cavero@uclm.es

**Keywords:** eosinophilic esophagitis, treatment, network meta-analysis, adults, children, glucocorticosteroids, budesonide, fluticasone, mometasone

## Abstract

**Background**: Swallowed topical corticosteroids (STCs) are used as the first-line therapy for eosinophilic esophagitis (EoE) and have been extensively studied in randomized controlled trials (RCTs); however, the presentation and doses varied widely among the studies. **Aim**: The goal of this study was to compare the safety and effectiveness of the different STC-based options in EoE patients. **Methods**: We performed a literature search for RCTs, spanning a time period from database inception to July 2024, in order to compare the efficacy and safety of all STCs used to induce or maintain EoE remission each other and also with placebo or proton pump inhibitors (PPIs) in a network meta-analysis. Outcomes are expressed as pooled risk ratios (RRs) of failure and 95% confidence intervals (CIs), and we aimed to evaluate histological remission at <15–20 eosinophils per high-power field (eos/hpf), <5–6 eos/hpf, and <1 eos/hpf. The effect sizes for symptomatic improvement and the mean differences for endoscopic EREFS improvement with 95% CIs were also measured. Adverse events were evaluated using RRs, and these included oropharyngeal and esophageal candidiasis and adrenal suppression. **Results**: Twenty studies involving 1455 patients with active EoE reported on STC effectiveness to induce remission; three additional studies on 232 patients assessed the maintenance of remission. Budesonide 1 mg orodispersible tablets ranked highest in SUCRA in terms of all histological remission endpoints. Budesonide from inhalation devices was the only option superior to placebo in improving symptoms. Budesonide viscous suspension was the only option superior to placebo in improving endoscopy. No therapy was significantly associated with the risk of any adverse event. Significant inconsistencies and small study effects were detected in multiple comparisons. **Conclusions:** Budesonide orodispersible tablets were the best option for achieving EoE histological remission, but not symptomatic or endoscopic improvement. STC formulations were as safe as placebo or PPI.

## 1. Introduction

Eosinophilic esophagitis (EoE) is an immune-mediated chronic type 2 inflammatory disorder of the esophagus [1], mainly triggered by exposure to food and, to a lesser extent, other environmental factors [2]. EoE is characterized clinically by a variety of symptoms of esophageal dysfunction and, histologically, by the infiltration of eosinophils in the esophageal mucosa [3,4].

Current first-line anti-inflammatory therapies for EoE consist of either the dietary elimination of culprit food antigens [5,6] or drug-based interventions consisting of proton pump inhibitors (PPIs) [7,8], topical corticosteroids [9], or dupilumab, a monoclonal antibody targeting Th2 cytokines [10]. From the first descriptions of EoE as a distinct disorder in the 1990s [11,12], cumulative research has reported on the effectiveness of these options to induce symptom remission and to reverse the inflammatory features in the esophageal biopsies of patients, especially the eosinophilic infiltration [13]. Additional treatment goals recently proposed include the improvement of esophageal endoscopic abnormalities and health-related quality of life [14].

Among first-line treatment options for EoE, topical corticosteroids with reduced bioavailability, swallowed instead of inhaled, provided variable histological remission rates in 30% to 90% of patients, depending on the formulas used [15,16]. The first evidence of the ability of these drugs to induce symptomatic improvement and control eosinophilic inflammation in young patients with EoE was reported in 1998 [17]. The effectiveness of swallowed topical corticosteroids (STCs) was confirmed throughout the following decade [18]: STCs applied in the patient’s mouth or pharynx, using metered dose inhalation systems while held in apnea and subsequently swallowing the product [19], gave way to viscous formulas that suspend or dissolve the drug to better reach the esophageal surface [20]. The variable composition of these formulas, the different drugs and doses used, and the changing volumes of suspension and drug concentration provided heterogeneous results [21].

The first randomized clinical trial (RCT) evaluating the capability of STCs to induce EoE remission was published in 2006 [22]. Since then, independent researchers and pharmaceutical companies have carried out an increasing number of RCTs, providing evidence that STCs overall are superior to placebo in achieving EoE histological and symptomatic improvement [15]. Three previous network meta-analyses (NMAs) compared the ability of STCs to induce EoE remission [23,24,25]: the earliest was conducted before most industry-sponsored trials were published, while the subsequent two included all pharmacological therapies for EoE evaluated through RCTs, including biologics. However, none of these NMAs examined all relevant histological thresholds for remission, performed subgroup analyses by drug formulation, assessed long-term therapy, or systematically graded the certainty of evidence.

As differences in efficacy and safety among the different presentations and active ingredients of STCs could exist, the present NMA aimed to compare the efficacy of the different STC formulations in both inducing and maintaining clinical and histological remission in patients with active EoE.

## 2. Materials and Methods

### 2.1. Search Strategy and Selection Criteria

This NMA followed the Preferred Reporting Items for Systematic Reviews incorporating Network Meta-Analysis (PRISMA-NMA) guidelines [26] (Tables S18 and S19) and the Cochrane Collaboration Handbook [27]. This study has been registered with The International Prospective Register of Systematic Reviews (PROSPERO), registration number CRD42015020362. Two independent authors (A.J.L. and A.A.) conducted the literature search through the PubMed, EMBASE, and Scopus databases during the period from inception to 24 July 2024. The reference lists of each previous systematic review were also screened for other relevant publications.

The literature search was performed by applying the following terms, Boolean operators and “Clinical Trials” filters: (topic steroids OR glucocorticoids OR fluticasone* OR budesonide OR beclometasone OR mometasone OR ciclesonide OR steroids OR swallowed) AND (eosinophilic esophagitis OR eosinophilic oesophagitis).

The titles and abstracts of potentially eligible studies were screened separately on the basis of the inclusion criteria by two reviewers (A.J.L. and I.R.-C.). The full texts of the selected studies were examined. Finally, the two reviewers assessed the reasons for excluding or including the studies. Disagreements were solved by consensus or with the intervention of a third researcher.

### 2.2. Inclusion and Exclusion Criteria

This meta-analysis included phase 2 and phase 3 RCTs, with no language restrictions. Studies evaluating the effects of different low-bioavailability corticosteroid drugs administered topically on outcomes in EoE patients were selected, regardless of the type of participants. Where two or more studies provided data on the same sample, the study presenting the most detailed results or with the largest sample size was chosen. Studies using any topical corticosteroid as an intervention (typically budesonide, fluticasone, or mometasone) were suitable for inclusion, as were studies comparing different types of corticosteroid drugs and examining EoE treatment with or without a placebo or active comparator (PPI or oral prednisone).

Patients concomitantly treated with food elimination diets or biological drugs were excluded, whereas those who were co-treated with PPI therapy were included if they had previously demonstrated non-response to PPIs based on esophageal biopsies. Duplicate patient datasets, including conference abstracts published prior to full-paper publication and laboratory studies evaluating the impact of STCs on non-clinical aspects of EoE, were also excluded. Authors were contacted for further clarification when required.

### 2.3. Data Extraction and Definition of Outcome Measures

Two reviewers (A.J.L. and A.A.) separately extracted data into a Microsoft Excel file (version 16.7; Microsoft Corporation, Redmond, WA, USA) and another two authors (I.R.-C. and C.A.-B.) confirmed the information. The following information on the included studies was abstracted by the two authors independently: title and reference details (first author, journal, year, country), study aim (induction or maintenance of remission), study population characteristics (number of patients, sex, age, EoE diagnostic criteria, concomitant PPI treatment, use of endoscopic dilation in the study period), intervention details (drug used, dose and drug delivery method in the exposure group) and comparators (placebo/other active drug), and treatment length. Data were extracted as intention-to-treat analyses, when available; all dropouts were assumed to be treatment failures.

The outcome data extracted included the following aspects, whenever available:

- Histological remission, assessed using several peak cut-off points of eosinophil counts: (a) a peak eosinophil count (PEC) of <15 or <20 eosinophils per high-power field (hpf), which is below the diagnostic infiltration threshold defined for EoE [3,28] and is recommended as the histological remission criterion for regular clinical practice [14]; (b) a PEC of <5 to <6 eos/hpf (histological remission criteria defined by the regulatory European Medicines Agency [EMS] and Food and Drug Administration [FDA] [29], respectively); and (c) the deep histological remission of <1 eos/hpf.
- Clinical improvement, as measured with either validated (the Dysphagia Symptom Questionnaire (DSQ) [30], the EoE activity index (EesAI) [31], or the 2-week or 30-day versions of the Mayo Dysphagia Questionnaire (MDQ) [32,33]) or non-validated scores (Dysphagia Symptom Score or DSS [34], Watson Dysphagia Scale [35], or a 0-to-10 numeric rating scale (NRS)).
- Changes induced by therapy in endoscopic features measured by the eosinophilic esophagitis endoscopic scoring system (EREFS system) [36].
- Histopathological changes in grade (severity) and stage (extent), all measured by the EoE histologic scoring system or EoEHSS [37].
- Quality of life, measured using the EoE-QoL-A questionnaire [38].
- Adverse events, including oral or pharyngeal candidiasis, esophageal candidiasis, and adrenal suppression.

### 2.4. Risk of Bias and Quality Assessment

Quality assessment was conducted independently by two authors (A.J.L. and I.C.-R.) [27]. The risk of bias of all RCTs was assessed using the Cochrane Collaboration’s tool for assessing risk of bias (RoB2) [39]. The overall bias was considered to be at “low risk of bias” when all domains were evaluated as “low risk”, at “some concerns” when at least one domain evaluated as “some concerns”, and at “high risk of bias” when at least one domain evaluated as “high risk” or when several domains were evaluated as “some concerns”. Disagreements were resolved by consensus or with the intervention of a third researcher (A.A.).

### 2.5. Statistical Analysis

Separate NMAs were performed to examine the three predefined therapeutic goals for histological remission. In addition, separate analyses were considered according to the following: (a) the active ingredient (regardless of the mode or vehicle of administering the drug within the esophagus); (b) the modality used to administer the drug (orodispersible tablets, viscous suspension or inhalation devices) regardless of the active ingredient; and (c) both criteria combined. Histological remission and adverse events results are presented as the pooled risk ratios (RRs) of failure to improve with 95% confidence intervals (95% CI), where if the RR is greater than 1 and the 95% CI does not cross 1, there is a significant benefit of one option over another or placebo. Clinical improvement results are presented as standardized mean differences and the effect sizes (ESs) measured for all the symptoms scales used in the different RCTs, and endoscopic improvement results based on EREFS are presented as mean differences (MDs).

Before carrying out the NMA, three assumptions were analyzed [27]: (1) Similarity was checked to verify that the studies included in the NMA were similar and comparable, thus avoiding bias between the comparisons, which could generate heterogeneity and inconsistency. Similarity was assessed by checking whether samples in each therapeutic intervention were similar in the baseline distribution of variables understood as effect modifiers (i.e., age, sex). (2) Homogeneity was checked for the nonexistence of heterogeneity between the results of the pairwise comparisons, ensuring that all study-related conditions were homogenous. The size and clinical relevance of heterogeneity was determined by the τ2 statistic, rated as low degree of clinical relevance (<0.04), moderate (0.04 to 0.14), or substantial (0.14 to 0.40) [40]. Finally, (3) consistency and transitivity were checked to ensure that no relevant discrepancies existed between direct and indirect evidence. The similarity assumption was determined using the node-splitting method [41].

The present NMA was conducted as follows: Firstly, we assessed the strength of the available evidence, using a network geometry graph to display the evidence for EoE outcomes. In this graph, the size of the nodes was proportional to the number of trial participants who received the intervention specified in the node, and the thickness of continuous line connecting nodes was proportional to the number of trials directly comparing the two treatments [42].

The comparative evaluation of the intervention effect on EoE outcomes was performed by conducting a random effects pairwise meta-analysis [27] and a frequentist network meta-analysis [42] for comparisons between interventions and control. We assessed heterogeneity using the I^2^ statistic [27], ranging from 0% to 100%. Based on the values of I^2^, we categorized heterogeneity as not important (0% to 30%), moderate (30% to 60%), substantial (60% to 75%), or considerable (75% to 100%). The corresponding *p* values were also considered.

Sensitivity analysis was conducted to evaluate the robustness of the pooled estimates, with a reanalysis performed by eliminating one study at a time.

The probability that each intervention was the most effective was presented using a rankogram. In addition, for each intervention, we estimated the surface under cumulative ranking (SUCRA) [43], which summarizes the information on the effect of each treatment into a single value. The best intervention had a SUCRA value of approximately 1, and the worst had a SUCRA value of approximately 0. SUCRA results are most meaningful when the difference in preference between consecutive ranks remains consistent across the entire rating scale.

Publication bias was tested using comparison-adjusted funnel plots and Egger’s regression asymmetry test [44], setting a threshold of <0.10 to indicate the presence of publication bias.

Analyses were performed using STATA 15 (StataCorp, College Station, TX, USA). Additionally, we performed a Bayesian network meta-analysis as a sensitivity analysis, using a gemtc of 0.8–2 and BUGSNET packages in R (version 4.0.2). The results were reported according to the Preferred Reporting Items for Systematic Reviews and Meta-Analyses extension statement for network meta-analyses [45].

### 2.6. Certainty of Evidence

We used the Grading of Recommendations, Assessment, Development, and Evaluation (GRADE) approach [46,47] to ascertain the certainty of evidence for the primary outcomes. The GRADE tool includes the following five distinct steps for each outcome: (1) allocate an a priori classification of “high” to RCTs and “low” to observational studies; (2) “downgrade” or “upgrade” the initial rating based on risk of bias, inconsistency, indirect evidence, imprecision, publication bias, large effect, dose–response relationship, and all plausible biases that only reduce an apparent treatment effect; (3) allocate the final rating of the quality of evidence as “high”, “moderate”, “low”, or “very low”; (4) address other influencing factors that affect the recommendation strength of recommendation for a course of action; and (5) make a “strong” or “weak” recommendation.

## 3. Results

### 3.1. Study Characteristics

**Figure 1:**
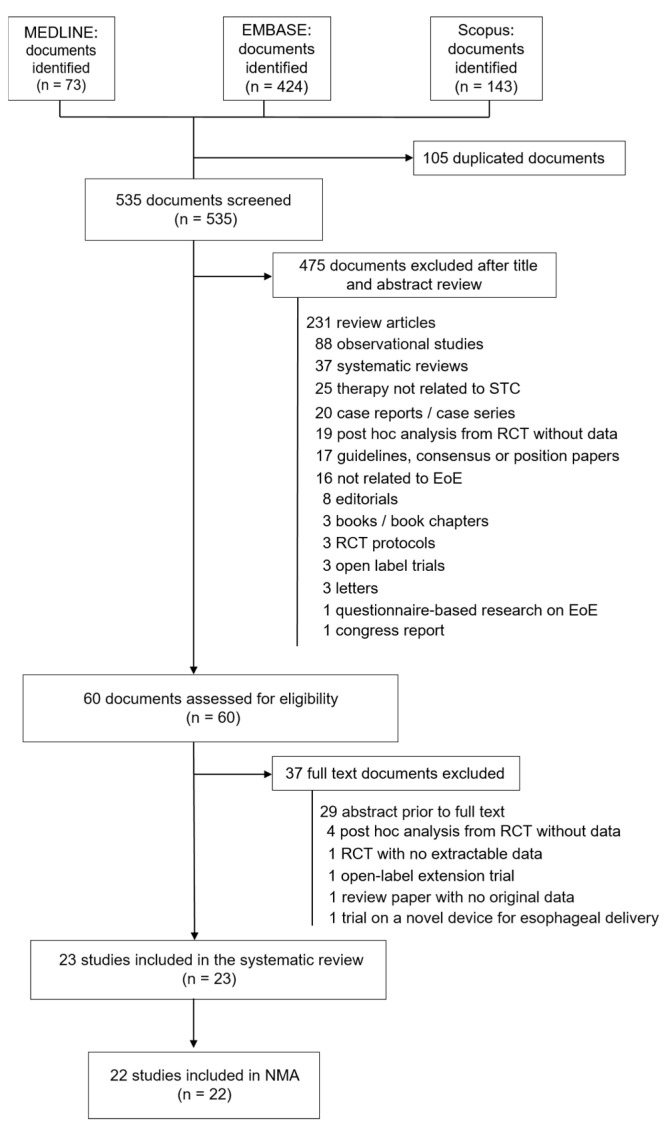
PRISMA flow diagram of study selection process.

After merging the search results from the different databases and removing duplicates, two independent reviewers assessed a total of 535 documents, of which 22 studies were included in our systematic review and NMA (Figure 1) [22,35,48,49,50,51,52,53,54,55,56,57,58,59,60,61,62,63,64,65,66]. One additional paper separately reported histopathological changes in one of these trials [67]. The studies were published between 2006 and 2024 and were conducted in ten different countries, with a large number performed in the United States of America. The sample size of the studies ranged from 24 to 318 patients. Excluded documents with reasons for exclusion are provided in Table S1.

**Figure 2:**
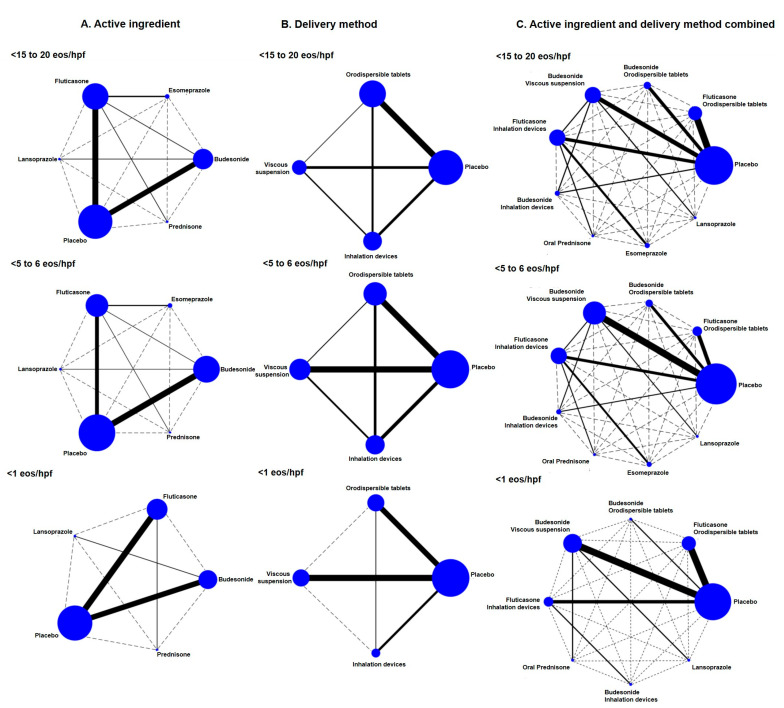
Network of available comparisons between different active ingredients (**A**), esophageal drug delivery methods (**B**), and both criteria combined (**C**) in the induction of histological remission of eosinophilic esophagitis, as defined by several histological outcomes. The size of the node is proportional to the number of trial participants, and the thickness of the continuous line connecting the nodes is proportional to the number of participants randomized in the trials directly comparing the two treatments.

A total of 19 studies involving 1455 subjects with active EoE reported on the effectiveness of STC to induce disease remission [22,48,49,50,51,52,53,54,55,56,57,58,59,60,61,62,63,65,66] (Figure 2). Three further studies involving 232 patients with inactive EoE analyzed the ability of STCs to maintain long-term remission [34,64,66]. The duration of interventions to induce EoE remission ranged from 2 to 14 weeks, and interventions to maintain remission lasted from 36 to 50 weeks.

Overall, 21 studies (including 19 for induction and the 3 for maintenance of remission) provided data on the therapeutic histological goal of PEC <15 to 20 eos/hpf, 20 studies included the goal of <5 to 6 eos/hpf, and 16 studies the goal of <1 eos/hpf. The main characteristics of the included studies are shown in Table 1.

### 3.2. Risk of Bias and GRADE

According to the Cochrane RoB2 tool, 56.5% of the studies raised some concerns regarding the risk of bias, with 39.1% and 4.3% studies showing low or high overall risks of bias, respectively. Traffic light plots and weighted bar plots risk-of-bias judgements within each bias domain [40] are shown in Figure S2A,B.

According to GRADE, the quality of evidence for each pairwise comparison and recommendation was classified as low for 25 comparisons (38.5%) and very low for 11 comparisons (16.9%) (Table S2).

### 3.3. Failure to Induce Histological Remission in EoE

For the histological remission endpoints, the NMA (lower diagonal) found that all the active STC ingredients, either budesonide (RR 0.03; 95% CI 0.01–0.09) or fluticasone (RR 0.03; 95% CI 0.01–0.11), as well as oral prednisone (RR 0.02; 95% CI <0.01–0.76) were more effective than the comparators at reducing PEC to <15–20 eos/hpf. Budesonide (RR 0.02; 95% CI 0.01–0.08), fluticasone (RR 0.03; 95% CI 0.01–0.11), and oral prednisone (RR 0.02; 95% CI <0.01–0.83) were also effective in reducing PEC to <5–6 eos/hpf. Finally, budesonide (RR 0.07; 95% CI 0.02–0.41), fluticasone (RR 0.03; 95% CI 0.01–0.14), and prednisone (RR 0.01; 95% CI <0.01–0.35) were superior to the comparators in reducing PEC to <1 eos/hpf (Table S3).

When the modality of drug administration was assessed, orodispersible tablets, viscous suspensions and inhalation devices were all significantly more effective than the comparators in achieving all the histological remission endpoints considered. In addition, orodispersible tablets were more effective than viscous suspensions in achieving <1 eos/hpf (Table S4).

**Figure 3:**
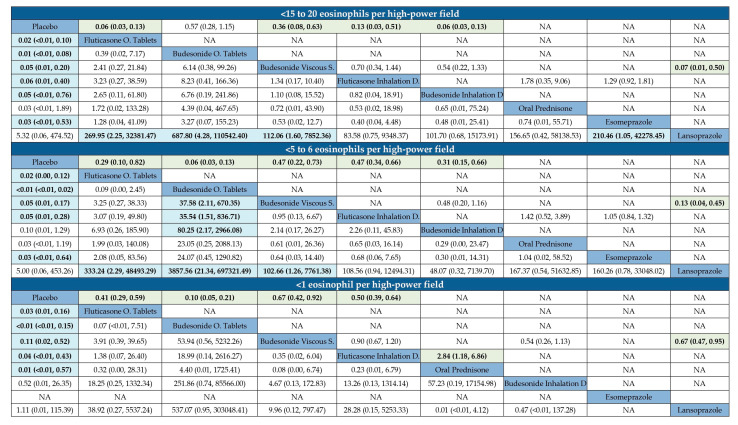
League tables presenting the comparative efficacy of various topical corticosteroid-based formulations and their comparators in inducing histological remission among patients with active eosinophilic esophagitis. Results are expressed as the likelihood of failure to induce histological remission, given as the risk ratio (RR) with 95% confidence intervals in league tables, and using different cut-off criteria of eosinophil density in esophageal biopsies to define remission. Upper right triangle gives the pooled RR from pairwise comparisons (column intervention relative to row), lower left triangle pooled RR from the network meta-analysis (row intervention relative to column). Values below 1 indicate a protective effect against the persistence of histological activity when the comparator intervention was used. Data in bold indicate statistically significant effects. NA: not available.

When both the active ingredient and the modality for delivering the drug into the esophagus criteria were combined, budesonide orodispersible tablets or BOT (RR 0.01; 95% CI <0.01–0.08; moderate certainty evidence), fluticasone orodispersible tablets (RR 0.02; 95% CI <0.01–0.10; moderate certainty evidence), budesonide inhalation devices (RR 0.05; 95% CI 0.00–0.76; moderate certainty evidence), fluticasone inhalation devices (RR 0.06; 95% CI 0.01–0.40; low certainty evidence), and budesonide viscous suspension (RR 0.05; 95% CI 0.01–0.20; very low certainty evidence) were all superior to comparators in reducing PEC to <15–20 eos/hpf (Table S2A). In addition, BOT (RR <0.01; 95% CI <0.01–0.02; low certainty evidence), fluticasone orodispersible tablets (RR 0.02; 95% CI <0.01–0.12: low certainty evidence), fluticasone inhalation devices (RR 0.05; 95% CI 0.01–0.28; low certainty evidence), and budesonide viscous suspension (RR 0.05; 95% CI 0.01–0.17; very low certainty evidence) were all effective in reducing PEC to <5–6 eos/hpf (Table S2B). Finally, BOT (RR <0.01; 95% CI <0.01–0.15; moderate certainty evidence), oral prednisone (RR 0.01; 95% CI <0.01–0.57), fluticasone orodispersible tablets (RR 0.03; 95% CI 0.01–0.16; low certainty evidence), fluticasone inhalation devices (RR 0.04; 95% CI <0.01–0.43; moderate certainty evidence), and budesonide viscous suspension (RR 0.11; 95% CI 0.02–0.52; very low certainty evidence) were all effective in achieving a PEC below 1 eos/hpf (Table S2C). Furthermore, BOT was more effective in achieving <1 eos/hpf than budesonide viscous suspension (Figure 3).

### 3.4. Effectiveness to Induce Symptomatic Improvement in EoE

**Figure 4:**
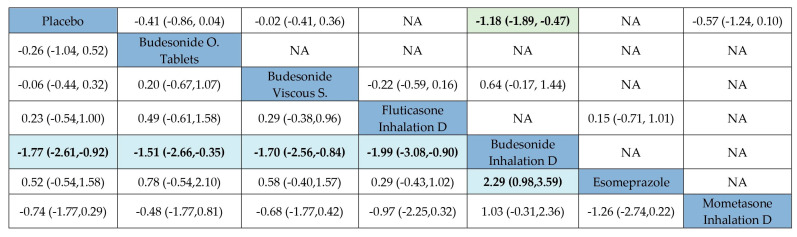
Comparative efficacy of different topical corticosteroid-based presentations versus comparators to induce clinical improvement in active eosinophilic esophagitis, expressed as standardized mean differences with their 95% confidence intervals in a league table. The upper right triangle gives the pooled standardized mean differences from pairwise comparisons (column intervention relative to row); the lower left triangle gives the pooled standardized mean differences from the network meta-analysis (row intervention relative to column). Data in bold indicate statistically significant effects. NA: Not available.

Regarding symptomatic improvement, the NMA estimates (lower diagonal) found that no active ingredient by itself could be considered significantly superior to placebo or esomeprazole (Table S5). When the mode used to administer the drug was considered, inhalation devices were found to be superior to placebo (ES −0.91; 95% CI −1.77, −0.06) (Table S6). Finally, when active ingredients and the method used to administer the drug were considered together, only budesonide inhalation devices showed significant superiority compared to placebo (high certainty evidence) or any other STC alternative (Figure 4 and Table S2D).

### 3.5. Failure to Achieve Endoscopic Improvement of Active EoE

We then examined the changes induced by therapy in EoE endoscopic features: Considering the network meta-analysis estimates (lower diagonal) for the active ingredient, budesonide induced a significant endoscopic improvement compared to placebo (MD −2.82; 95% CI −4.18, −1.46) (Table S7). For the modality of drug administration, viscous suspension showed significant superiority compared to placebo (MD −3.09; 95% CI −5.10, −1.07) (Table S8). When both criteria were combined, budesonide viscous suspension was the only option that was statistically superior to placebo (MD −3.09; 95% CI −5.10, −1.07; high-quality evidence) (Tables S2E and S9).

### 3.6. Efficacy According to Rankograms and SUCRA Values

Regarding active ingredients, oral prednisone showed the highest SUCRA for the histological remission endpoints of PEC < 15–20 eos/hpf (73%), <5–6 eos/hpf (73%), and <1 eos/hpf (95%) (Table S10).

When drug administration modality was considered, orodispersible tablets showed the highest SUCRA for histological remission defined either as PEC < 15–20 eos/hpf (85%), <5–6 eos/hpf (88%), or <1 eos/hpf (95%) (Table S11). In contrast, inhalation devices exhibited the highest SUCRA value (86%) for achieving symptomatic improvement. Finally, viscous suspensions had the highest SUCRA values (75%) for reducing endoscopic EoE features.

When active ingredient and modality to administer the drug were considered together, BOT performed better than the remaining treatments in achieving histological remission, defined either as PEC < 15–20 eos/hpf (SUCRA 88%), <5–6 eos/hpf (SUCRA 97%), or <1 eos/hpf (SUCRA 90%) (Table 2); budesonide inhalation devices ranked the highest for clinical improvement (SUCRA 99%) (Table S12) and budesonide viscous suspension for endoscopic improvement (76%) (Table S13).

### 3.7. Adverse Events of Treatment to Induce Active EoE Remission

**Figure 5:**
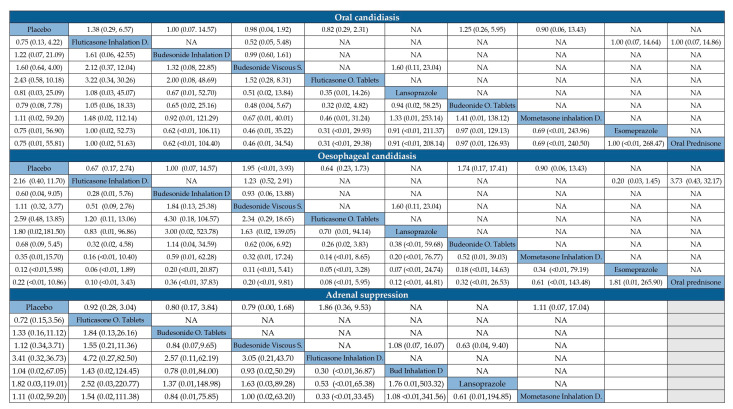
League tables comparing the risk of adverse events between different topical corticosteroid-based formulations and comparators during induction of remission therapy in patients with active eosinophilic esophagitis. Results are expressed as risk ratios (RRs) with 95% confidence intervals in league tables. Upper right triangle gives the pooled RR from pairwise comparisons (column intervention relative to row); lower left triangle gives the pooled RR from the network meta-analysis (row intervention relative to column). Values greater than 1 indicate a protective effect against the persistence of histological activity when the comparator intervention was used. NA: Not available.

The adverse events considered were oral candidiasis, esophageal candidiasis, and adrenal suppression. Considering the network meta-analysis estimates (lower diagonal), no statistically significant association was found between any active ingredient (Table S14), the method of administering the drug (Table S15), or the combination of both criteria (active ingredient and method) and, in terms of comparator alternatives, the risk of any adverse event (Figure 5 and Table S2F–H).

### 3.8. Sensitivity Analysis and Publication Bias

In the sensitivity analysis, neither the pooled RRs for histological remission and adverse events results nor the ES for clinical improvement or the MD for endoscopic features changed significantly when individual study data points were removed, one at a time, from any pairwise comparison analysis. Furthermore, considerable heterogeneity was found for budesonide versus placebo for histological remission defined as PEC <15– 20 eos/hpf (I^2^ 78.6, τ2 0.34), clinical improvement (I^2^ 77.0, τ2 0.13), and endoscopic improvement measured with EREFS score (I2 80.6, τ2 0.15). Heterogeneity was also high in the comparison of viscous suspension to placebo for histological remission defined as PEC < 15–20 eos/hpf (I^2^ 79.0, τ2 0.17) and for budesonide viscous suspension to placebo for PEC < 15–20 eos/hpf (I^2^ 79.0, τ2 0.17) (Tables S16 and S17).

In addition, there was evidence of a small study effect in the Egger test for multiple comparisons, including (A) fluticasone versus placebo for histological remission defined either as PEC < 15–20 eos/hpf (*p* = 0.011), <5–6 eos/hpf (*p* < 0.001) or <1 eos/hpf (*p* = 0.018) and risks for oral candidiasis, esophageal candidiasis, and adrenal suppression (*p* = 0.002, *p* < 0.001 and *p* < 0.001, respectively); (B) budesonide versus placebo for histological remission defined as an PEC < 15–20 eos/hpf (*p* = 0.001), endoscopic improvement (*p* = 0.021), risk for oral candidiasis (*p* = 0.006), esophageal candidiasis (*p* = 0.029), and adrenal suppression (*p* = 0.066, respectively); (C) inhalation devices versus placebo for histological remission defined as PEC < 15–20 eos/hpf (*p* = 0.056), oral candidiasis (*p* = 0.045), esophageal candidiasis (*p* = 0.012), and adrenal suppression (*p* = 0.094); (D) viscous suspension versus placebo for histological remission defined as PEC <5–6 eos/hpf (*p* = 0.053), endoscopic improvement (*p* = 0.030), oral candidiasis (*p* = 0.018), esophageal candidiasis (*p* = 0.098), and adrenal suppression (*p* = 0.092); (E) orodispersible tablets versus placebo for histological remission as PEC < 15–20 eos/hpf (*p* = 0.049) and <5–6 eos/hpf (*p* = 0.006), oral candidiasis (*p* = 0.001), and adrenal suppression (*p* = 0.077); (F) budesonide viscous suspension versus placebo for histological remission defined as PEC <5–6 eos/hpf (*p* = 0.053), reduction in EREFS score (*p* = 0.030), oral candidiasis (*p* = 0.018), esophageal candidiasis (*p* = 0.098), and adrenal suppression (*p* = 0.092); (G) fluticasone orodispersible tablets versus placebo for histological remission as PEC <5–6 eos/hpf (*p* = 0.001), oral candidiasis (*p* = 0.001), and esophageal candidiasis (*p* < 0.001); and (H) fluticasone inhalation devices versus placebo for esophageal candidiasis (*p* = 0.090).

### 3.9. Maintenance of EoE in Remission

Finally, three RCTs involving 280 patients assessed the effectiveness of STCs to maintain EoE in long-term remission by comparing budesonide with a placebo [34,64,66]. The doses administered ranged from 0.5 to 4 mg daily, using inhalation devices [34], oral suspension [66], or orodispersible tablets [64]. In the oldest trial, 0.25 mg twice-daily swallowed budesonide from an inhalation suspension device was no more effective than the placebo at maintaining EoE in clinical–histological remission over 50 weeks [34]. Administering an oral suspension of 2 mg budesonide twice daily provided non-significantly higher chances than placebo in maintaining EoE in clinical–histological remission over 36 weeks in the ITT analysis [66]. Finally, BOT at twice-daily doses of either 0.5 mg or 1 mg maintained EoE in clinical–histological remission over 48 weeks in 75% and 73% patients, respectively (compared to 4.4% in the placebo group), and was also superior at maintaining endoscopic remission and quality of life.

In terms of safety, esophageal or oropharyngeal candidiasis was documented in 5.1% and 1.7% of patients administered budesonide, but no patients administered placebo. Adrenal suppression was suspected in 5.6% and 0% of patients assigned to the budesonide and placebo groups, respectively.

## 4. Discussion

To our knowledge, this network meta-analysis is the largest and most comprehensive effort to summarize and compare the effectiveness and safety of STCs in treating patients with EoE. SCTs were the first drugs used to treat EoE [17]. Despite the fact that only two STC-based treatments are currently on the market in certain countries, they constitute the standard treatment for many patients. EoE has represented a challenge for topical treatment throughout the three decades since this disease was first characterized, and STCs are the most extensively tested treatments in terms of number of patients and RCTs. Pairwise meta-analyses already demonstrated their superiority over placebo [9,69], but the results were highly heterogeneous due to the variety of drugs used, their doses and concentrations, and especially how they were applied to ensure adequate contact time with the esophageal mucosa [70]. For the first time, we analyzed the effectiveness of the different STCs for EoE based on their active ingredients and vehicles for administration, as well as both variables combined. Thus, we aimed to provide evidence of the role that the drug delivery method plays in the effectiveness of a topical therapy for EoE.

Overall, our analyses demonstrate that the different active ingredients and formulations were all generally superior to placebo at inducing the histological remission of EoE. However, the more stringent the histological remission criteria considered, the more important it was to select an appropriate combination of active ingredients and pharmacological vehicles. Thus, BOT was the most effective alternative in indirect comparisons for achieving a PEC < 1 eos/hpf, while also showing superiority over oral viscous or inhaled budesonide presentations. BOT also had the highest SUCRA values for all histological outcomes considered; thus, our results confirm observations of previous NMAs that compared drug therapies to treat EoE beyond STCs [24,25] by including biologics, which are not currently recommended as a first-line therapy for EoE [71].

This trend, however, was not observed for other efficacy endpoints, such as the symptomatic improvement or reversal of endoscopic features. The symptom measurement instruments used in RCTs varied widely, from disease-specific scales in the more recent studies, to numerical rating scores and standard instruments for dysphagia in the earlier ones, the latter having proven insufficient to discriminate among the symptoms of EoE [52,72]. We combined results from different measurement instruments using standardized mean differences, as effect size was the only acceptable method, but we could not demonstrate that any alternative was superior to another or to a placebo. In contrast, the only previous NMA that evaluated drug-induced symptomatic improvement in EoE found BOT to be superior to all the other interventions. This was after homogenizing the results of different studies as RR, which is methodologically questionable. However, BOT was the most effective alternative for endoscopic improvement based on the EREFS score.

Regarding safety, our study found no differences in the main risks associated with SCT treatment compared to placebo or PPI treatment. However, due to the evidence of the small study effect shown in multiple comparisons, these results must be taken with caution.

Three RCTs provided data on the ability of STC to maintain EoE remission with topical corticosteroids, all using budesonide as an active ingredient. However, the wide range of doses (from 0.5 to 2 mg twice daily) and the different vehicles prevented comparisons. More studies are needed to clearly determine the best long-term treatment of EoE, especially in terms of safety.

The present study has several strengths. Firstly, it is the first PROSPERO-registered NMA on therapy for EoE, providing clinicians with pre-defined STC outcomes in the management of EoE. Secondly, a range of histological outcomes were considered, comprising those that are clinically meaningful in both normal clinical practice and in therapeutic research [14]. Thirdly, the risk of bias was systematically evaluated in all source documents using Cochrane’s RoB-2 tool, with the strength of our results being assessed using sensitivity analyses. GRADE was applied to assess the certainty of the evidence for all the outcomes in pairwise comparisons by considering all important clinical and methodological domains. Finally, this NMA includes the largest number of individual trials and patients treated with STCs for EoE to date.

One of the limitations of our study is that we included studies in the NMA that were carried out over a two decades, resulting in a large amount of heterogeneity. This was due to the variability in how the histological effects of interventions were assessed across the studies. The eosinophil threshold for EoE diagnosis decreased during this period from 24 to 15 eos/hpf. As an hpf is not a standardized measure and varies among microscope manufacturers, we considered both measures to be equivalent. The same was decided for <5 and <6 eos/hpf. Both cut-offs were defined as relevant therapeutic endpoints for EoE remission in a recent core outcome document [14]. Additionally, the response to symptoms was evaluated even more heterogeneously across studies, which prevented us from demonstrating the benefits of one therapy over another. Furthermore, the inconsistency of many of our comparisons and the presence of small size study effects meant that we had to view our results cautiously, and, as changes in histological and quality of life scores were exceptionally evaluated, we were unable to provide results on these. All budesonide suspensions or orodispersible tablets were merged as single interventions, despite variable doses (1 to 4 mg daily) or treatment lengths (from 2 to 12 weeks) being assessed. We do not believe that stratifying by study duration could have made a notable difference given that the most effective therapies for inducing remission were those of shorter duration (6 and 2 weeks in studies with BOT) [56,59]. Finally, the limited number of pediatric trials [22,48,49,55] and the absence of separate data from studies involving adolescents [34,51,54,57,62,66,68] prevented us from independently evaluating younger patient groups.

## 5. Conclusions

In summary, this NMA supports the efficacy of budesonide and fluticasone over placebo for inducing the histological remission of EoE independently of the formulation used to administer the drug. However, BOT performed better than the other treatments, including oral prednisone and esomeprazole, in achieving histological remission defined at any PEC threshold. For symptomatic improvement, only budesonide inhalation devices were superior to other corticosteroid-based alternatives and achieved the highest SUCRA; however, this result should be taken with caution due to limitations in instruments to measure EoE symptoms. Finally, budesonide viscous suspension was the only option that was superior to placebo and ranked above the other options in terms of improving endoscopic EoE features. All the therapeutic options compared were safe, with no increased risk of esophageal or oropharyngeal candidiasis compared to placebo. However, the high level of inconsistency in the sensitivity analysis and the small study effect detected in multiple comparisons prevented us from establishing a solid hierarchy for STC-based therapies for EoE, although our finding that BOT is the most effective therapy for achieving histological remission overall reproduces the results of previous NMAs and increases their plausibility.

## Figures and Tables

**Table 1:** Characteristics of included studies of randomized controlled trials of topical steroids with the aim of inducing or maintaining remission in patients with active EoE.

First Author, Year	Study Period	Country	Sites	Population	Study Type	N (Male)	Drug	Formulation	Daily Dose	Daily Intakes	Comparator	Treatment Duration	Co-Therapy Allowed *
Konikoff, 2006 [22]	January 2003 to August 2005	USA	Single center	Children	Induction	36 (26)	Fluticasone	Metered dose inhaler	880 mcg	2	Placebo	12 weeks	PPI
Schaefer, 2008 [48]	February 2000 to November 2004	USA	Single center	Children and adolescents	Induction	80 (59)	Fluticasone	Metered dose inhaler	800 mcg for ages 1–10 1660 mcg for ages ≥ 11 years	4	Prednisone	4 weeks	No
Dohil, 2010 [49]	February 2008 to July 2009	USA	Single center	Children	Induction	24 (20)	Budesonide	Oral viscous suspension + lansoprazole	1 mg or 2 mg	1	Placebo + lansoprazole	12 weeks	PPI
Peterson, 2010 [50]	January 2005 to October 2006	USA	Single center	Adults	Induction	30 (23)	Fluticasone	Metered dose inhaler	880 mcg	2	Esomeprazole	8 weeks	No
Straumann, 2010 [51]	May 2006 to April 2007	Switzerland	Single center	Children and adults	Induction	36 (31)	Budesonide	Metered dose inhaler	2 mg	2	Placebo	2 weeks	PPI
Straumann, 2011 [34]	June 2006 to May 2007	Switzerland	Single center	Children and adults	Maintenance	28 (24)	Budesonide	Metered dose inhaler	0.5 mg	2	Placebo	50 weeks	PPI
Alexander, 2012 [52]	October 2005 to December 2009	USA	Single center	Adults	Induction	42 (30)	Fluticasone	Metered dose inhaler	1760 mcg	2	Placebo	6 weeks	PPI
Dellon, 2012 [60]	March 2010 to May 2011	USA	Single center	Children	Induction	25 (15)	Budesonide	Oral viscous suspension	2 mg	2	Budesonide metered dose inhaler	8 weeks	NA
Moawad, 2013 [53]	April 2008 to October 2010	USA	Single center	Adults	Induction	42 (38)	Fluticasone	Metered dose inhaler	880 mcg	2	Esomeprazole	8 weeks	PPI
Butz, 2014 [54]	December 2006	USA	Multicenter	Children and adults	Induction	42 (35)	Fluticasone	Metered dose inhaler	1760 mcg	2	Placebo	12 weeks	PPI
Gupta, 2015 [55]	NA	USA	Multicenter	Children	Induction	71 (57)	Budesonide	Oral viscous suspension	0.35 to 0.5 mg	1	Placebo	12 weeks	PPI
									1.4 to 2 mg	1	Placebo		
									2.8 to 4 mg	2	Placebo		
Miehlke, 2016 [56]	June 2011 to April 2013	3 European countries	Multicenter	Adults	Induction	76 (63)	Budesonide	Orodispersible tablets	2 mg	2	Placebo	2 weeks	PPI
								Orodispersible tables	4 mg	2			
								Oral viscous	4 mg	2			
Dellon, 2017 [57]	July 2012 to October 2014	USA	Multicenter	Children and adults	Induction	93 (64)	Budesonide	Oral viscous suspension	4 mg	2	Placebo	12 weeks	PPI
Dellon, 2019 [58]	2014 to 2018	USA	Single-center	Adults	Induction	111 (74)	Budesonide/ Fluticasone	Oral viscous suspension	2 mg	2	Fluticasone	8 weeks	PPI
Collins, 2019 [68]	NA	USA	Multicenter	Children and adolescents	Induction	93	Budesonide	Oral viscous suspension	2 mg	2	Placebo	24 weeks	PPI
Lucendo, 2019 [59]	November 2015 to October 2016	Six European Countries	Multicenter	Adults	Induction	88 (70)	Budesonide	Orodispersible tablets	2 mg	2	Placebo	6 weeks	PPI
Hirano, 2020 [65]	October 2011 to October 2012	USA	Multicenter	Children and adults	Induction	24 (15)	Fluticasone	Orodispersible tablets	1.5 mg	2	Placebo	8 weeks	PPI
									3 mg	1			
Straumann, 2020 [64]	2016 to 2019	Six European Countries	Multicenter	Adults	Maintenance	204 (169)	Budesonide	Orodispersible tablet	1 mg	2	Placebo	48 weeks	PPI
									2 mg				
Tytor et al. 2021 [61]	April 2014 to August 2019	Sweden	Multicenter	Adults	Induction	36 (33)	Mometasone	Metered dose inhaler	800 mcg	4	Placebo	8 weeks	NA
Hirano, 2022 [63]	2015 to 2019	USA	Multicenter	Children and adults	Induction	318	Budesonide	Oral Viscous suspension	4 mg	2	Placebo	12 weeks	PPI
Dellon, 2022 [66]	May 2017 to August 2018	USA, Canada, 4 European countries	Multicenter	Adults	Induction	103 (70)	Fluticasone	Orodispersible tablets	6 mg	2	Placebo	14 weeks	PPI
									3 mg	1			
									3 mg	2			
									1.5 mg	1			
Dellon et al. 2022 [69]	2016 to 2019	USA	Multicenter	Children and adults	Maintenance	48 (30)	Budesonide	Oral viscous suspension	4 mg	2	Placebo	36 weeks	PPI
Lucendo et al. 2024 [63]	2019 to 2022	8 European countries and Turkey	Multicenter	Children and adolescents	Induction	100 (76)	Budesonide	Oral viscous suspension	0.5 mg	1	Placebo	12 weeks	PPI
									1 mg	2			
									1 mg	1			
									2 mg	2			

Abbreviations: PPI proton pump inhibitors; NA, not available. * Response PPI treatment was previously excluded.

**Table 2:** Effectiveness ranking of different topical corticosteroid-based presentations and comparators to induce histological remission of active eosinophilic esophagitis, defined by different cut-off criteria of eosinophil density in esophageal biopsies.

**<15 to 20 Eosinophils per High-Power Field**					
	**Rank Statistics**			**Probabilities**	
	**Mean**	**Median**	**95% CIs**	**Best**	**SUCRA**
Budesonide orodispersible tablets	2.0	7.0	1.0–9.0	0.52	0.88
Fluticasone orodispersible tablets	3.0	6.0	1.0–8.0	0.13	0.75
Oral prednisone	4.2	3.0	2.0–6.0	0.16	0.60
Esomeprazole	3.6	4.0	2.0–5.0	0.14	0.67
Budesonide inhalation devices	4.9	4.0	1.0–5.0	0.05	0.51
Budesonide viscous suspension	5.2	6.0	2.0–7.0	0.01	0.48
Fluticasone inhalation devices	5.3	6.0	1.0–7.0	0.00	0.47
Placebo	8.1	9.0	1.0–9.0	0.00	0.11
Lansoprazole	8.7	8.0	1.0–8.0	0.00	0.04
**<5 to 6 Eosinophils per High-Power Field**					
	**Rank Statistics**			**Probabilities**	
	**Mean**	**Median**	**95% CIs**	**Best**	**SUCRA**
Budesonide orodispersible tablets	1.2	7.0	1.0–9.0	0.85	0.97
Fluticasone orodispersible tablets	3.0	6.0	1.0–7.0	0.06	0.75
Oral prednisone	4.1	4.0	2.0–5.0	0.06	0.61
Esomeprazole	4.1	4.0	2.0–5.0	0.04	0.61
Fluticasone inhalation devices	4.7	5.0	1.0–6.0	0.00	0.53
Budesonide viscous suspension	5.1	5.0	1.0–7.0	0.00	0.49
Budesonide inhalation devices	5.9	4.0	1.0–6.0	0.00	0.39
Placebo	8.1	8.0	1.0–9.0	0.00	0.11
**<1 Eosinophil per High-Power Field**					
	**Rank Statistics**			**Probabilities**	
	**Mean**	**Median**	**95% CIs**	**Best**	**SUCRA**
Budesonide orodispersible tablets	1.6	5.0	1.0–8.0	0.67	0.92
Oral prednisone	2.3	4.0	1.0–6.0	0.27	0.81
Fluticasone orodispersible tablets	3.1	4.0	1.0–7.0	0.04	0.69
Fluticasone inhalation devices	3.6	4.0	1.0–6.0	0.01	0.63
Budesonide viscous suspension	5.1	4.0	1.0–7.0	0.00	0.42
Budesonide inhalation devices	6.5	4.0	2.0–6.0	0.00	0.22
Placebo	6.9	7.5	1.0–8.0	0.00	0.16
Lansoprazole	6.9	5.0	1.0–7.0	0.00	0.15

## Data Availability

The authors confirm that the data supporting the findings of this study are available within the article and its Supplementary Materials.

## Supplementary Materials

The following supporting information can be downloaded at: https://www.mdpi.com/article/10.3390/jcm14217823/s1, Figure S1. Risk of bias of randomized controlled trials included in the systematic review according to the Cochrane RoB2 tool. (A), ‘Traffic light’ plots of the domain-level judgments for each individual result; (B), Weighted bar plots of the distribution of risk of bias judgements within each bias domain. Figure S2. Network of available comparisons between different active ingredients (A), esophageal drug delivery methods (B), and both criteria combined (C) in risk for developing adverse events, consisting in oral and esophageal candidiasis and adrenal suppression, in patients with eosinophilic esophagitis. The size of the node is proportional to number of trial participants, and thickness of the continuous line connecting nodes is proportional to number of participants randomized in trials directly comparing the two treatments. Table S1. Excluded Studies After Full Paper Revision and Reason for Exclusion. Table S2A–H. Quality grading of evidence for effectiveness and safety or STC in patients with eosinophilic esophagitis. Table S3. Comparative efficacy of different topical corticosteroids with low-bioavailability, oral prednisone, proton pump inhibitors and placebo to induce histological remission in patients with active eosinophilic esophagitis. Table S4. Comparative efficacy of different modalities to administer topical corticosteroids or placebo to induce histological remission of active eosinophilic esophagitis. Table S5. Comparative efficacy of different topical corticosteroids with low-bioavailability, oral prednisone, proton pump inhibitors and placebo to induce clinical improvement in active eosinophilic esophagitis, expressed as standardized mean differences with their 95% confidence intervals in league tables. Table S6. Comparative efficacy of different modalities to administer topical corticosteroids or placebo to induce clinical improvement in active eosinophilic esophagitis, expressed as standardized mean differences with their 95% confidence intervals in league tables. Table S7. Comparative efficacy of different active ingredients of topical corticosteroids versus comparators for changes induced by therapy in endoscopic features of eosinophilic esophagitis measured by the EREFS system as mean differences with their 95% confidence intervals in league tables. Table S8. Comparative efficacy of different modalities to administer topical corticosteroids or placebo to induce endoscopic improvement measured by EREFS score in patients with active eosinophilic esophagitis, system as mean differences with their 95% confidence intervals in league tables. Table S9. Comparative efficacy of different topical corticosteroids-based presentations versus comparators for changes induced by therapy in endoscopic features of eosinophilic esophagitis measured by the EREFS system as mean differences with their 95% confidence intervals in league tables. Table S10. Effectiveness ranking of different active ingredients, irrespective of delivery method, proton pump inhibitors and placebo to induce histological remission of active eosinophilic esophagitis, defined by different cut-off criteria of eosinophil density in esophageal biopsies. Table S11. Effectiveness ranking of different methods for esophageal delivery of topical corticosteroids and placebo to induce histological remission of active eosinophilic esophagitis, defined by different cut-off criteria of eosinophil density in esophageal biopsies. Table S12. Effectiveness ranking of different presentations of topical corticosteroids versus comparators to induce clinical improvement in active eosinophilic esophagitis. Table S13. Effectiveness ranking of different presentations of topical corticosteroids versus comparators to endoscopic features measured by EREFS score in active eosinophilic esophagitis. Table S14. Comparative risk of different active ingredients of swallowed topical corticosteroids versus comparators in the risk of adverse events resulting from induction of remission therapy in patients with active eosinophilic esophagitis. Table S15. Comparative risk of different esophageal delivery methods for swallowed topical corticosteroids, irrespective of active ingredients versus comparators in the risk of adverse events resulting from induction of remission therapy in patients with active eosinophilic esophagitis. Table S16. Heterogeneity statistics for each comparison for different: A. active ingredient; B. esophageal drug delivery modality; and C. combination of both criteria on histological remission, defined by several cut-off points. Table S17. Heterogeneity statistics for each comparison for different: A. active ingredient; B. esophageal drug delivery modality; and C. combination of both criteria on clinical improvement. Table S18. PRISMA-checklist. Table S19. PRISMA-abstract-checklist.

## Abbreviations

The following abbreviations are used in this manuscript:

EoE	Eosinophilic esophagitis
Eos	eosinophils
EREFS	Eosinophilic esophagitis endoscopic scoring system
hpf	High-power field
NA	Not applicable/Not available
NMA	Network meta-analysis
MD	Mean differences
PEC	Peak eosinophil count
PPI	Proton pump inhibitor
RCT	Randomized controlled trial
RR	Risk ratio
STC	Swallowed topical corticosteroid
SUCRA	Surface under the cumulative ranking curve
