# Supplementary material for: Comparative Efficacy and Safety of Swallowed Topical Corticosteroids in Eosinophilic Esophagitis: A Network Meta-Analysis
Source: J Clin Med. 2025 Nov 4;14(21):7823. doi: 10.3390/jcm14217823 (PMC12610317; doi:10.3390/jcm14217823)
Supplement: Supplementary file 1 [file jcm-14-07823-s001.zip › jcm-3919639-supplementary-1.pdf]

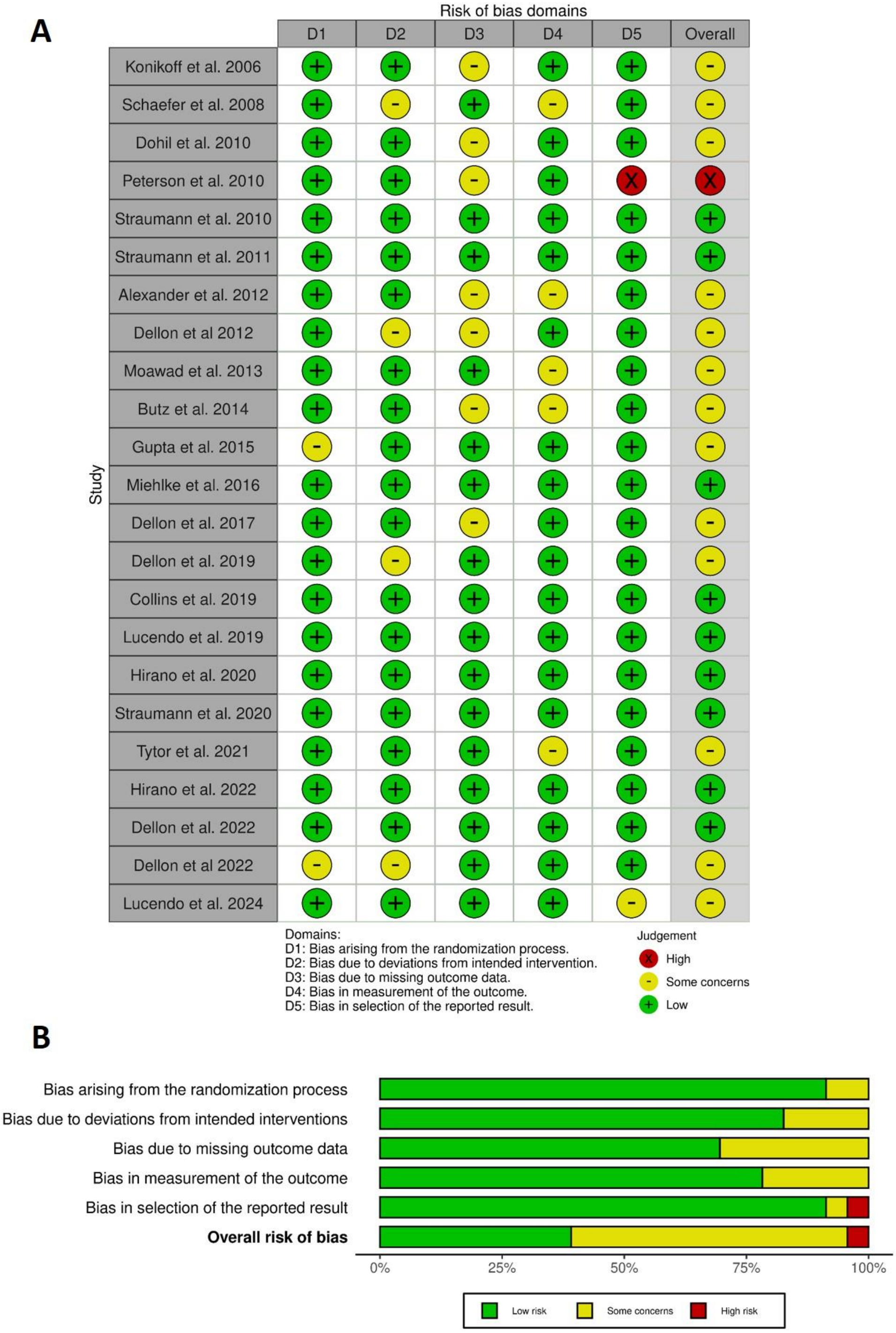

Figure S1. Risk of bias of randomized controlled trials included in the systematic review according to the Cochrane RoB2 tool. (A), 'Traffic light' plots of the domain-level judgments for each individual result; (B), Weighted bar plots of the distribution of risk of bias judgements within each bias domain.

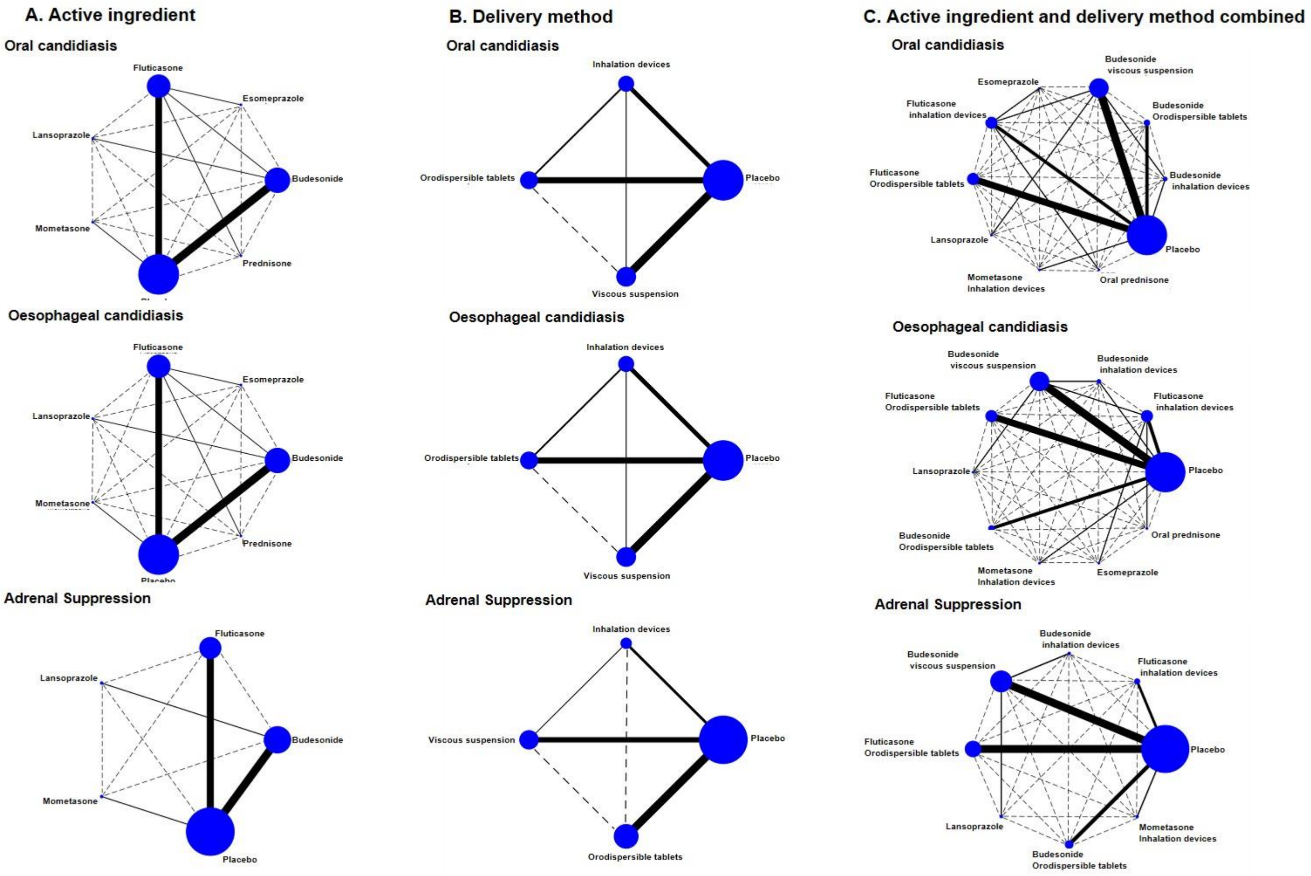

Figure S2. Network of available comparisons between different active ingredients (A), esophageal drug delivery methods (B), and both criteria combined (C) in risk for developing adverse events, consisting in oral and esophageal candidiasis and adrenal suppression, in patients with eosinophilic esophagitis. The size of the node is proportional to number of trial participants, and thickness of the continuous line connecting nodes is proportional to number of participants randomized in trials directly comparing the two treatments.

Table S1. Excluded Studies After Full Paper Revision and Reason for Exclusion

Reference	Reason for Exclusion
Biedermann, L., Lucendo, A. J., Miehlke, S., Schlag, C., Santander Vaquero, C., Ciriza De Los Rios, C., Hartmann, D., Madisch, A., Hruz, P., Hayat, J., Von Arnim, U., Bredenoord, A., Müller, R., Greinwald, R., Schoepfer, A., Attwood, S. E., & Straumann, A. (2019). A novel budesonide orodispersible tablet formulation is highly effective to maintain endoscopic inflammatory remission and even complete endoscopic remission in adult patients with eosinophilic esophagitis: Results from the 48-weeks, double-blind, placebo-controlled pivotal EOS-2 trial. United European Gastroenterology Journal, 7(8), 55-55. https://doi.org/10.1177/205064061985467	Conference abstract previous to full paper, already included
Collins, M., Dellon, E., Hirano, I., Katzka, D., Mukkada, V., Williams, J., Lan, L., & Desai, N. (2019). Baseline characterization of adolescent and adult patients with eosinophilic esophagitis (EOE) using the eoe histology scoring system: Analyses from a randomized, placebo-controlled trial. Journal of Pediatric Gastroenterology and Nutrition, 69. https://doi.org/10.1097/MPG.0000000000002518	Conference abstract previous to an already included study
Collins, M. H., Dellon, E. S., Hirano, I., Katzka, D. A., Williams, J., Lan, L., & Desai, N. K. (2019). Baseline characterization of patients with eosinophilic esophagitis (EOE) using the eoe histology scoring system and correlations with endoscopic and symptom outcomes: Analyses from a randomized, placebo-controlled trial. American Journal of Gastroenterology, 114, S230-S230. https://doi.org/10.14309/01.ajg.0000591100.32919.d0	Conference abstract previous to an already included study
Collins, M. H., Hirano, I., Katzka, D. A., Mukkada, V. A., Falk, G. W., Williams, J. E., Desai, N. K., Lan, L., & Dellon, E. S. (2020). Improved Histopathologic Features in Patients with Eosinophilic Esophagitis: Results and Analyses from a Phase 3, Randomized, Placebo-Controlled Trial of Budesonide Oral Suspension. American Journal of Gastroenterology, 115(SUPPL), S200-S201. https://doi.org/10.14309/01.ajg.0000703664.24718.78	Conference abstract with data of an already included study
Collins, M., Hirano, I., Katzka, D., Cianferoni, A., Williams, J., Desai, N., Lan, L., & Dellon, E. (2020). Budesonide Oral Suspension Improves Histologic Features In Patients With Eosinophilic Esophagitis: Results From A Phase 3, Randomized, Double-blind, Placebo-Controlled Trial. Journal of Allergy and Clinical Immunology, 145(2), AB166-AB166. https://doi.org/10.1016/j.jaci.2019.12.341	Conference abstract with data of an already included study
Dellon, E. S., Collins, M. H., Katzka, D. A., Hudgens, S., Lan, L., Williams, J., Vera-Llonch, M., & Hirano, I. (2021). Improvements in Dysphagia and Pain With Swallowing in Patients With Eosinophilic Esophagitis Receiving Budesonide Oral Suspension. Clinical Gastroenterology and Hepatology, 19(4), 699-706.e4. https://doi.org/10.1016/j.cgh.2020.03.060	Conference abstract previous to an already included study
Dellon, E. S., Collins, M. H., Katzka, D. A., Mukkada, V. A., Falk, G. W., Goodwin, B., Wojtowicz, A. M., Boules, M., Zhang, W., Desai, N. K., & Hirano, I. (2021). TIME TO FIRST DYSPHAGIA SYMPTOM RESPONSE IN PATIENTS WITH	Sub-analysis of data already reported in an included study

EOSINOPHILIC ESOPHAGITIS: POST HOC ANALYSIS FROM A PHASE 3 TRIAL OF BUDESONIDE ORAL SUSPENSION. Gastroenterology, 160(6), S-249-S-250. https://doi.org/10.1016/S0016-5085(21)01337-8	
Dellon, E. S., Falk, G. W., Hirano, I., Lucendo, A., Schlag, C., Schoepfer, A. M., Eagle, G., Nezamis, J. P., Sparling, N., Richardson, P., Knoop, K., & Paty, J. (2020). AN EPISODE-BASED, PATIENT-REPORTED OUTCOME MEASURE OF DYSPHAGIA ACCURATELY CAPTURES RESPONSE TO TREATMENT WITH APT-1011 IN A PHASE 2B, RANDOMIZED, DOUBLE-BLIND, PLACEBO-CONTROLLED, CLINICAL TRIAL IN ADULTS WITH EOSINOPHILIC ESOPHAGITIS. Gastroenterology, 158(6), S-817. https://doi.org/10.1016/S0016-5085(20)32722-0	Sub-analysis of data already reported in an included study
Dellon, E. S., Falk, G. W., Lucendo, A., Schlag, C., Schoepfer, A. M., Eagle, G., Nezamis, J. P., Comer, G. M., & Knoop, K. (2021). FLUTICASONE PROPIONATE ORALLY DISINTEGRATING TABLET (APT-1011) IMPROVES HISTOLOGIC, ENDOSCOPIC AND SYMPTOMATIC RESPONSES IN EOSINOPHILIC ESOPHAGITIS PATIENTS WITH FIBRO-STENOTIC FEATURES: RESULTS FROM A PHASE 2B TRIAL. Gastroenterology, 160(6), S-111-S-112. https://doi.org/10.1016/S0016-5085(21)01014-3	Conference abstract with data of an already included study
Dellon, E. S., Falk, G. W., Lucendo, A., Schlag, C., Schoepfer, A. M., Eagle, G., Nezamis, J. P., Comer, G. M., Knoop, K., & Hirano, I. (2021). Low Rates Of Glucocorticoid-Related Adverse Effects With Long-Term Treatment Of Eosinophilic Esophagitis With Fluticasone Propionate Orally Disintegrating Tablet (Apt-1011): Results From 52 Weeks Of Exposure In A Phase 2b Trial. Gastroenterology, 160(6), S-248-S-249. https://doi.org/10.1016/S0016-5085(21)01335-4	Conference abstract with data of an already included study
Dellon, E. S., Katzka, D. A., Collins, M. H., Gupta, S. K., Lan, L., Williams, J., & Hirano, I. (2019). Safety and Efficacy of Budesonide Oral Suspension Maintenance Therapy in Patients With Eosinophilic Esophagitis. Clinical Gastroenterology and Hepatology, 17(4), 666-673.e8. https://doi.org/10.1016/j.cgh.2018.05.051	Open-label extension of a trial, with double-blind phase data reported in an already included study
Dellon, E. S., Katzka, D. A., Collins, M. H., & Hirano, I. (2015). The dysphagia symptom questionnaire is responsive and valid for measuring patient-reported outcomes in eosinophilic esophagitis: Analysis from a multicenter randomized placebo controlled trial. Gastroenterology, 148(4), S792-S792.	Sub-analysis of data already reported in an included study
Dellon, E. S., Lucendo, A., Schlag, C., Schoepher, A., Falk, G. W., Richardson, P., Eagle, G., Nezamis, J., Comer, G., Knoop, K., & Hirano, I. (2020). Safety and Efficacy of Long-Term Treatment of EoE with Fluticasone Propionate Orally Disintegrating Tablet (APT-1011): Results from 40 Weeks Treatment in a Phase 2b Randomized Double-Blind Placebo-Controlled Trial. American Journal of Gastroenterology, 115(SUPPL), S169-S170. https://doi.org/10.14309/01.ajg.0000703428.23468.41	Conference abstract with data of an already included study
Dellon, E. S., Lucendo Villarin, A., Schlag, C., Schoepfer, A., Knoop, K., Richardson, P., Falk, G., Eagle Taran, G., Comer, G. M., & Hirano, I. (2019). Safety and efficacy of 4 dosage regimens of APT-1011: Results from a phase 2B randomized double-blind placebo-controlled trial to select the best dosage regimen for further development. (Clinicaltrials.gov NCT03191864;	Conference abstract with data of an already included study

EUDRACT number: 2016-004749-10). United European Gastroenterology Journal, 7(10), 1420-1420. https://doi.org/10.1177/2050640619888859	
Dellon, E. S., Woosley, J. T., Arrington, A., McGee, S. J., Covington, J., Moist, S. E., Gebhart, J. H., Tylicki, A. E., Shoyoye, S. O., Martin, C., & al., et. (2019a). ORAL VISCOUS BUDESONIDE VERSUS SWALLOWED FLUTICASONE INHALER FOR INITIAL TREATMENT OF ADOLESCENTS AND ADULTS WITH EOSINOPHILIC ESOPHAGITIS: a RANDOMIZED, DOUBLE-BLIND, DOUBLE-DUMMY CLINICAL TRIAL. Gastroenterology, 156(6), S-72-S-73. https://doi.org/10.1016/S0016-5085(19)36966-5	Conference abstract with data of an already included study
Falk, G., Hirano, I., Lucendo, A. J., Schoepfer, A. M., Schlag, C., Eagle, G., Nezamis, J., Comer, G. M., Knoop, K., & Dellon, E. S. (2021). Time to clinically meaningful improvement in dysphagia frequency in adults with eosinophilic esophagitis: A post-hoc analysis from a phase 2b trial of APT-1011, a fluticasone propionate oral disintegrating tablet. American Journal of Gastroenterology, 116(SUPPL), S152-S153. https://doi.org/10.14309/01.ajg.0000773884.45924.08	Conference paper with a sub-analysis of data from an already included study
Goodwin, B., Williams, J., Desai, N. K., & Lan, L. (2020a). PGI1 ESTIMATION OF THE MINIMAL IMPORTANT DIFFERENCES IN EOSINOPHILIC ESOPHAGITIS QUALITY OF LIFE (EOE-QOL): ANALYSIS FROM A PHASE 3, RANDOMIZED, PLACEBO-CONTROLLED TRIAL OF BUDESONIDE ORAL SUSPENSION. Value in Health, 23, S143-S143. https://doi.org/10.1016/j.jval.2020.04.361	Sub-analysis of data from an already included study, which provides no results on changes of QoL induced by therapy
Goodwin, B., Williams, J., Desai, N. K., & Lan, L. (2020b). PGI36 ESTIMATION OF THE MINIMAL IMPORTANT DIFFERENCES IN EUROQOL-5 DIMENSIONS (EQ-5D) FOR PATIENTS WITH EOSINOPHILIC ESOPHAGITIS: ANALYSIS FROM A PHASE 3, RANDOMIZED, PLACEBO-CONTROLLED TRIAL OF BUDESONIDE ORAL SUSPENSION. Value in Health, 23, S149-S149. https://doi.org/10.1016/j.jval.2020.04.390	Sub-analysis of data from an already included study, which provides no results on changes of QoL induced by therapy
Gupta, S. K., Collins, M. H., Lewis, J. D., & Farber, R. H. (2011). Efficacy and safety of oral budesonide suspension (OBS) in pediatric subjects with eosinophilic esophagitis (EoE): Results from the double-blind, placebo-controlled PEER study. Gastroenterology, 140(5), S179-S179. https://doi.org/10.1016/S0016-5085(11)60725-7	Conference abstract previous to an already included study
Gupta, S., Mukkada, V., Dellon, E., Gold, B., Collins, M., Katzka, D., Falk, G., Lan, L., Desai, N., Williams, J., & Hirano, I. (2021). Efficacy And Safety Of Budesonide Oral Suspension In A Pediatric Population: Pooled Data From A Phase 2 And Phase 3 Trial In Patients With Eosinophilic Esophagitis. Journal of Allergy and Clinical Immunology, 147(2), AB91-AB91. https://doi.org/10.1016/j.jaci.2020.12.348	Pooled data from 2 studies already included in the review

Hirano, I., Collins, M. H., Katzka, D. A., Mukkada, V. A., Falk, G. W., Williams, J., Desai, N. K., Lan, L., & Dellon, E. S. (2020a). 227 BUDESONIDE ORAL SUSPENSION (BOS) IMPROVES ENDOSCOPIC ACTIVITY IN ADOLESCENTS AND ADULTS WITH EOSINOPHILIC ESOPHAGITIS: RESULTS AND CORRELATION ANALYSIS FROM A PHASE 3, RANDOMIZED, PLACEBO-CONTROLLED TRIAL. *Gastroenterology*, *158*(6), S-42-S-43. https://doi.org/10.1016/S0016-5085(20)30788-5	Conference abstract previous to an already included study
Hirano, I., Collins, M. H., Katzka, D. A., Mukkada, V. A., Falk, G. W., Williams, J., Desai, N. K., Lan, L., Morey, R., & Dellon, E. S. (2019). Efficacy of budesonide oral suspension for eosinophilic esophagitis in adolescents and adults: Results from a phase 3, randomized, placebo-controlled trial. American Journal of Gastroenterology, 114, S205-S206. https://doi.org/10.14309/01.ajg.0000590932.88962.0b	Conference abstract previous to an already included study
Hirano, I., Collins, M., Katzka, D., Mukkada, V., Falk, G., Johnston, D., Soteres, D., Williams, J., Desai, N., Lan, L., Morey, R., & Dellon, E. (2019). BUDESONIDE ORAL SUSPENSION TREATMENT IMPROVES DYSPHAGIA SYMPTOMS IN PATIENTS WITH EOSINOPHILIC ESOPHAGITIS. Annals of Allergy, Asthma and Immunology, 123(5), S56-S57. https://doi.org/10.1016/j.anai.2019.08.338	Conference abstract with data of an already included study
Hirano, I., Katzka, D. A., Collins, M. H., & Dellon, E. S. (2015). Randomized, double-blind, placebo controlled trial demonstrates the efficacy of oral budesonide suspension in improving endoscopically identified esophageal abnormalities in eosinophilic esophagitis. Gastroenterology, 148(4), S29-S29.	Conference abstract previous to an already included study
Hirano, I., Schoepfer, A. M., Comer, G. M., Safroneeva, E., Meltzer, B. A., & Falk, G. W. (2017). A randomized, double-blind, placebo-controlled trial of a fluticasone propionate orally disintegrating tablet in adult and adolescent patients with eosinophilic esophagitis: A phase 1/2A safety and tolerability study. Gastroenterology, 152(5), S195-S195.	Conference abstract previous to an already included study
Lucendo, A., Miehlke, S., Vieth, M., Schlag, C., Biedermann, L., Santander, C., Ciriza de los Ríos, C., Hartmann, D., Madisch, A., Hruz, P., Hayat, J. O., Arnim, U. V., Bredenoord, A. J., Schubert, S., Attwood, S. E., Mueller, R., Greinwald, R., Schoepfer, A. M., & Straumann, A. (2019). BUDESONIDE ORODISPERSIBLE TABLETS ARE HIGHLY EFFECTIVE TO MAINTAIN CLINICO-HISTOLOGICAL REMISSION IN ADULT PATIENTS WITH EOSINOPHILIC ESOPHAGITIS: RESULTS FROM THE 48-WEEKS, DOUBLE-BLIND, PLACEBO-CONTROLLED, PIVOTAL EOS-2 TRIAL. Gastroenterology, 156(6), S-1509. https://doi.org/10.1016/S0016-5085(19)40852-4	Conference abstract previous to an already included study
Lucendo, A., Miehlke, S., Vieth, M., Schlag, C., Von Arnim, U., Molina-Infante, J., Hartmann, D., Bredenoord, A. J., De Los Ríos, C. C., Schubert, S., Brückner, S., Madisch, A., Hayat, J. O., Tack, J. F., Attwood, S. E., Mueller, R., Greinwald, R., Schoepfer, A. M., & Straumann, A. (2017). Budesonide orodispersible tablets are highly effective for treatment of active eosinophilic esophagitis: Results from a randomized, double-blind, placebo-controlled, pivotal multicenter trial (EOS-1). Gastroenterology, 152(5), S207-S207.	Conference abstract previous to an already included study

Lucendo AJ, Nantes-Castillejo Ó, Straumann A, Biedermann L, Bredenoord AJ, Guagnozzi D, Blas-Jhon L, Wiechowska-Kozlowska A, Weidlich S, von Arnim U, Santander-Vaquero C, Perelló A, Pérez-Martínez I, Barrio J, Vieth M, Gouya G, Dellon ES. Clinical Trial: Safety and Efficacy of a Novel Oesophageal Delivery System for Topical Corticosteroids Versus Placebo in the Treatment of Eosinophilic Oesophagitis. Aliment Pharmacol Ther. 2025 Feb;61(3):444-455. doi: 10.1111/apt.18443	Proof-of-concept trial on a device for controlled oesophageal release of medication, which used mometasone furoate in patients with eosinophilic esophagitis
Miehlke, S., Lucendo, A. J., Vieth, M., Schlag, C., Von Arnim, U., Molina-Infante, J., Hartmann, D., Bredenoord, A. J., Ciriza De Los Rios, C., Schubert, S., Brückner, S., Madisch, A., Hayat, J., Tack, J., Attwood, S., Mueller, R., Greinwald, R., Schoepfer, A., & Straumann, A. (2017). A randomized, double-blind, placebocontrolled, pivotal multicenter trial with budesonide orodispersible tablets for treatment of active eosinophilic esophagitis (EOS-1). Allergy: European Journal of Allergy and Clinical Immunology, 72, 78-79. https://doi.org/10.1111/all.13250	Conference abstract with data of an already included study
Moawad, F., Dias, J., Veerappan, G., Baker, T., Maydonovitch, C., Wong, R. (2011). Comparison of aerosolized swallowed fluticasone to esomeprazole for the treatment of eosinophilic esophagitis. American Journal of Gastroenterology, 106, S12-S12.	Conference abstract with data of an already included study
Oliva, S., Ruggiero, C., Rossetti, D., Tinti, V., Salimbene, L., Sarli, M., Alessio, R., Papoff, P., Tiberti, A., & Cucchiara, S. (2018). A randomized controlled trial comparing six-food elimination diet vs topical steroids in inducing and maintaining remission of paediatric eosinophilic esophagitis. Journal of pediatric gastroenterology and nutrition, 66, 89-.	Patients were scoped for assessment of effectiveness after 14 week off treatment
Safroneeva, E., Schoepfer, A. M., & Dellon, E. S. (2020). STRONG ASSOCIATION BETWEEN DYSPHAGIA FREQUENCY WITH DAILY OR WEEKLY RECALL IN ADULTS WITH EOSINOPHILIC ESOPHAGITIS: ANALYSIS OF TWO SYMPTOM-BASED PATIENT-REPORTED OUTCOME MEASURES USED IN A COMPARATIVE EFFICACY RANDOMIZED TRIAL. Gastroenterology, 158(6), S-819. https://doi.org/10.1016/S0016-5085(20)32726-8	Sub-analysis of data already reported in an included study
Schlag, C., Biedermann, L., Lucendo, A. J., Miehlke, S., Santander Vaquero, C., Ciriza De Los Rios, C., Hartmann, D., Madisch, A., Hruz, P., Hayat, J., Von Arnim, U., Bredenoord, A., Müller, R., Greinwald, R., Schoepfer, A., Attwood, S. E., & Straumann, A. (2019). Budesonide orodispersible tablets are superior to maintain and even further improve quality of life in adult patients with eosinophilic esophagitis: Results from the 48-weeks, double-blind, placebo-controlled pivotal EOS-2 trial. United European Gastroenterology Journal, 7(8), 705-705. https://doi.org/10.1177/205064061985467	Conference abstract with data of an already included study

Straumann, A., & Hruz, P. (2009). What's new in the diagnosis and therapy of eosinophilic esophagitis? Current Opinion in Gastroenterology, 25(4), 366-371.	Review paper with no original data provided
Straumann, A., Lucendo, A. J., Greinwald, R., Mueller, R., & Attwood, S. (2017). Efficacy and safety of budesonide orodispersible tablets in active eosinophilic oesophagitis: Results from a randomised, double-blind, placebo-controlled, pivotal, European multicentre trial (EOS-1). Swiss Medical Weekly, 147, 10S-10S.	Conference abstract with data of an already included study
Straumann, A., Lucendo Villarin, A. J., Miehlke, S., Vieth, M., Schlag, C., Von Arnim, U., Molina-Infante, J., Hartmann, D., Bredenoord, A. J., Ciriza De Los Rios, C., Schubert, S., Brueckner, S., Madisch, A., Hayat, J., Tack, J., Schoepfer, A., Greinwald, R., Mueller, R., & Attwood, S. (2017). Efficacy and safety of budesonide orodispersible tablets in active eosinophilic oesophagitis: Results from a randomised, doubleblind, placebo-controlled, Pivotal, European multicenre trial (EOS-1). United European Gastroenterology Journal, 5(5), A146-A147. https://doi.org/10.1177/2050640617725668	Conference abstract with data of an already included study
Turner, R., Rohay, J. M., Sparling, N., Whitsett, J., Eagle, G., Nezamis, J., Knoop, K., Dellon, E. S., Hirano, I., & Paty, J. (2021). Patient-reported symptom improvement following treatment with APT-1011 in patients with eosinophilic esophagitis as measured by the prose: Results from flute, a phase 2b clinical trial. American Journal of Gastroenterology, 116(SUPPL), S203-S203. https://doi.org/10.14309/01.ajg.0000774304.21136.36	Conference abstract with data of an already included study

Table S2A. Quality grading of evidence for treatment modality (combining active ingredient with oesophageal delivery method) in achieving histological remission eosinophilic esophagitis defined as <15-to-20 eosinophils per high power field.

Certainty assessment							№ of patients		Effect		Certainty	Importance
**№ of studies**	**Study design**	**Risk of bias**	**Inconsistency**	**Indirectness**	**Imprecision**	**Other considerations**	**Treatment**	**Treatment/Placebo**	**Relative (95% CI)**	**Absolute (95% CI)**		
**Fluticasone Orodispersible tablets vs. Placebo**												
**6**	Randomised trials	Not serious	Not serious	Not serious	Serious[a]	None	100	92	-	SMD **0.26 SD higher.** (0.18 higher. to 0.4 higher.)	⊕⊕⊕◯ Moderate	IMPORTANT
**Budesonide Orodispersible tablets vs. Placebo**												
**3**	Randomised trials	Not serious	Not serious	Not serious	Serious[a]	None	97	67	-	SMD **0.06 SD higher.** (0.03 higher. to 0.13 higher.)	⊕⊕⊕◯ Moderate	CRITICAL
**Budesonide Viscous suspension vs. Placebo**												
**5**	Randomised trials	Serious[b]	Very serious [c]	Not serious	Serious[a]	None	328	203	-	SMD **0.36 SD higher.** (0.08 higher. to 0.63 higher.)	⊕◯◯◯ Very low	CRITICAL
**Buesonide Viscous suspension vs. Lansoprazole**												
**1**	Randomised trials	Serious[b]	-	Not serious	Serious[a]	None	15	9	-	SMD **0.07 SD higher.** (0.01 higher. to 0.5 higher.)	⊕⊕◯◯ Low	IMPORTANT
**Fluticasone Inhalation devices vs. Placebo**												
**3**	Randomised trials	Serious[b]	Serious[d]	Not serious	Serious[a]	None	70	50	-	SMD **0.53 SD higher.** (0.29 higher. to 0.95 higher.)	⊕◯◯◯ Very low	NOT IMPORTANT
**Budesonide Viscous suspension vs. Budesonide Inhalation devices**												
**1**	Randomised trials	Serious[b]	-	Not serious	Not serious	None	12	13	-	SMD **0.54 SD higher.** (0.29 higher. to 0.95 higher.)	⊕⊕⊕◯ Moderate	IMPORTANT
**Fluticasone Inhalation devices vs. Oral Prednisone**												
**1**	Randomised trials	Serious[b]	-	Not serious	Not serious	None	36	32	-	SMD **1.78 SD higher.** (0.35 higher. to 9.06 higher.)	⊕⊕⊕◯ Moderate	NOT IMPORTANT
**Fluticasone Inhalation devices vs. Esomeprazole**												
**2**	Randomised trials	Very serious [e]	Not serious	Not serious	Not serious	None	36	36	-	SMD **1.29 SD higher.** (0.92 higher. to 1.81 higher.)	⊕⊕◯◯ Low	NOT IMPORTANT
**Budesonide Viscous suspension vs. Fluticasone Inhalation devices**												
**1**	Randomised trials	Serious[b]	-	Not serious	Not serious	None	56	55	-	SMD **0.7 SD higher.** (0.34 higher. to 1.44 higher.)	⊕⊕⊕◯ Moderate	NOT IMPORTANT
**Budesonide Inhalation devices vs. Placebo**												
**1**	Randomised trials	Not serious	-	Not serious	Serious[a]	None	18	18	-	SMD **0.13 SD higher.** (0.03 higher. to 0.51 higher.)	⊕⊕⊕◯ Moderate	NOT IMPORTANT

CI: Confidence intervals; **SMD:** Standardized mean difference. a. The direction of the effect is controversial; b. Some concerns risk of bias; c. Considerable heterogeneity; d. Substantial heterogeneity; e. High risk of bias

Table S2B. Quality grading of evidence for treatment modality (combining active ingredient with oesophageal delivery method) in achieving histological remission eosinophilic esophagitis defined as <5-to-6 eosinophils per high power field.

Certainty assessment							№ of patients		Effect		Certainty	Importance
**№ of studies**	**Study design**	**Risk of bias**	**Inconsistency**	**Indirectness**	**Imprecision**	**Other considerations**	**Treatment**	**Treatment/Placebo**	**Relative (95% CI)**	**Absolute (95% CI)**		
**Fluticasone Inhalation devices vs. Placebo**												
**3**	Randomised trials	Serious[a]	Not serious	Not serious	Serious[b]	None	70	50	-	SMD **0.47 SD higher.** (0.34 higher. to 0.66 higher.)	⊕⊕◯◯ Low	NOT IMPORTANT
**Budesonide Viscous suspension vs. Placebo**												
**9**	Randomised trials	Serious[a]	Very serious[c]	Not serious	Serious[b]	None	272	194	-	SMD **0.47 SD higher.** (0.22 higher. to 0.73 higher.)	⊕◯◯◯ Very low	CRITICAL
**Budesonide Orodispersible tablets vs. Placebo**												
**3**	Randomised trials	Not serious	Not serious	Not serious	Serious[b]	Publication bias[d]	97	67	-	SMD **0.06 SD higher.** (0.03 higher. to 0.13 higher.)	⊕⊕◯◯ Low	CRITICAL
**Fluticasone Inhalation devices vs. Oral Prednisone**												
**1**	Randomised trials	Serious[a]	-	Not serious	Not serious	None	36	32	-	SMD **1.42 SD higher.** (0.52 higher. to 3.89 higher.)	⊕⊕⊕◯ Moderate	NOT IMPORTANT
**Fluticasone Orodispersible tablets vs. Placebo**												
**4**	Randomised trials	Not serious	Serious[c]	Not serious	Serious[b]	Publication bias[d]	84	76	-	SMD **0.29 SD higher.** (0.1 higher. to 0.82 higher.)	⊕◯◯◯ Very low	NOT IMPORTANT
**Fluticasone Inhalation devices vs. Esomeprazole**												
**2**	Randomised trials	Very serious[e]	Not serious	Not serious	Not serious	None	36	36	-	SMD **1.05 SD higher.** (0.84 higher. to 1.32 higher.)	⊕⊕◯◯ Low	NOT IMPORTANT
**Budesonide Viscous suspension vs. Lansoprazole**												
**1**	Randomised trials	Serious[a]	-	Not serious	Serious[b]	None	15	9	-	SMD **0.13 SD higher.** (0.04 higher. to 0.45 higher.)	⊕⊕◯◯ Low	NOT IMPORTANT
**Budesonide Inhalation devices vs. Placebo**												
**1**	Randomised trials	Not serious	-	Not serious	Not serious	None	18	18	-	SMD **0.31 SD higher.** (0.15 higher. to 0.66 higher.)	⊕⊕⊕⊕ High	NOT IMPORTANT
**Budesonide Viscous suspension vs. Budesonide Inhalation devices**												
**1**	Randomised trials	Serious[a]	-	Not serious	Serious[b]	None	12	13	-	SMD **0.48 SD higher.** (0.2 higher. to 1.16 higher.)	⊕⊕◯◯ Low	NOT IMPORTANT

CI: Confidence intervals; **SMD:** Standardized mean difference. a. Some concerns risk of bias; b. The direction of the effect is controversial; c. Considerable heterogeneity; d. Publication bias; e. High risk of bias

Table S2C. Quality grading of evidence for treatment modality (combining active ingredient with oesophageal delivery method) in achieving histological remission eosinophilic esophagitis defined as <1 eosinophil per high power field.

Certainty assessment							№ of patients		Effect		Certainty	Importance
**№ of studies**	**Study design**	**Risk of bias**	**Inconsistency**	**Indirectness**	**Imprecision**	**Other considerations**	**Treatment**	**Treatment/Placebo**	**Relative (95% CI)**	**Absolute (95% CI)**		
**Fluticasone Orodispersible tablets vs Placebo**												
**6**	Randomised trials	Serious[a]	Not serious	Not serious	Not serious	Publication bias[b]	100	92	-	SMD **0.41 SD higher.** (0.29 higher. to 0.59 higher.)	⨁⨁◯◯ Low	NOT IMPORTNT
**Budesonide Orodispersible tablets vs Placebo**												
**1**	Randomised trials	Serious[a]	-	Not serious	Not serious	None	59	29	-	SMD **0.1 SD higher.** (0.05 higher. to 0.21 higher.)	⨁⨁⨁◯ Moderate	CRITICAL
**Budesonide Viscous suspension vs Placebo**												
**6**	Randomised trials	Serious[a]	Very serious [c]	Not serious	Not serious	None	311	196	-	SMD **0.67 SD higher.** (0.42 higher. to 0.92 higher.)	⨁◯◯◯ Very low	CRITICAL
**Fluticasone Inhalation devices vs Placebo**												
**3**	Randomised trials	Serious[a]	Not serious	Not serious	Not serious	None	70	50	-	SMD **0.5 SD higher.** (0.39 higher. to 0.64 higher.)	⨁⨁⨁◯ Moderate	NOT IMPORTANT
**Fluticasone Inhalation devices vs Budesonide Viscous suspension**												
**1**	Randomised trials	Serious[a]	-	Not serious	Not serious	None	55	56	-	SMD **0.9 SD higher.** (0.67 higher. to 1.2 higher.)	⨁⨁⨁◯ Moderate	NOT IMPORTANT
**Fluticasone Inhalation devices vs Oral Prednisone**												
**1**	Randomised trials	Serious[a]	-	Not serious	Serious[d]	None	36	32	-	SMD **2.84 SD higher.** (1.18 higher. to 6.86 higher.)	⨁⨁◯◯ Low	NOT IMPORTANT
**Budesonide Inhalation devices vs Budesonide Viscous suspension**												
**1**	Randomised trials	Serious[a]	-	Not serious	Serious[d]	None	13	12	-	SMD **0.54 SD higher.** (0.26 higher. to 1.13 higher.)	⨁⨁◯◯ Low	NOT IMPORTANT
**Budesonide Viscous suspension vs Lansoprazole**												
**1**	Randomised trials	Serious[a]	-	Not serious	Serious[d]	None	15	9	-	SMD **0.67 SD higher.** (0.47 higher.	⨁⨁◯◯ Low	IMPORTANT

									to 0.95 higher.)			

Table S2D. Quality grading of evidence for treatment modality (combining active ingredient with oesophageal delivery method) in achieving symptomatic remission of eosinophilic oesophagitis.

Certainty assessment							№ of patients		Effect		Certainty	Importance
**№ of studies**	**Study design**	**Risk of bias**	**Inconsistency**	**Indirectness**	**Imprecision**	**Other considerations**	**Treatment**	**Treatment/Placebo**	**Relative (95% CI)**	**Absolute (95% CI)**		
**Fluticasone Inhalation devices vs Esomeprazole**												
**2**	Randomised trials	Very serious [a]	Serious[b]	Not serious	Not serious	None	36	36	-	SMD **0.15 SD higher.** (0.71 lower to 1.01 higher.)	⊕◯◯◯ Very low	NOT IMPORTANT
**Budesonide Viscous suspension vs Fluticasone Inhalation devices**												
**1**	Randomised trials	Serious[c]	Not serious	-	Serious[d]	None	56	55	-	SMD **0.22 SD lower** (0.59 lower to 0.16 higher.)	⊕⊕◯◯ Low	NOT IMPORTANT
**Budesonide Inhalation devices vs Placebo**												
**1**	Randomised trials	Not serious	Not serious	-	Not serious	None	18	18	-	SMD **1.18 SD lower** (1.89 lower to 0.47 lower)	⊕⊕⊕⊕ High	NOT IMPORTANT
**Budesonide Viscous suspension vs Placebo**												
**3**	Randomised trials	Serious[c]	Serious[b]	Not serious	Not serious	None	309	184	-	SMD **0.02 SD lower** (0.41 lower to 0.36 higher.)	⊕⊕◯◯ Low	CRITICAL
**Budesonide Orodispersible tablets vs Placebo**												
**1**	Randomised trials	Not serious	Not serious	-	Not serious	None	59	29	-	SMD **0.41 SD lower** (0.86 lower to 0.04 higher.)	⊕⊕⊕⊕ High	CRITICAL
**Mometasone Inhalation devices vs Placebo**												
**1**	Randomised trials	Serious[c]	-	Not serious	Not serious	None	17	19	-	SMD **0.57 SD lower** (1.24 lower to 0.1 higher.)	⊕⊕⊕◯ Moderate	NOT IMPORTANT
**Budesonide Viscous suspension vs Budesonide Inhalation devices**												
**1**	Randomised trials	Serious[c]	-	Not serious	Serious[d]	None	12	13	-	SMD **0.64 SD higher.** (0.17 lower to 1.44 higher.)	⊕⊕◯◯ Low	IMPORTANT

Table S2E. Quality grading of evidence for treatment modality (combining active ingredient with oesophageal delivery method) in achieving endoscopic improvement of eosinophilic esophagitis, according to EREFS score.

Certainty assessment							№ of patients		Effect		Certainty	Importance
№ of studies	Study design	Risk of bias	Inconsistency	Indirectness	Imprecision	Other considerations	Treatment	Treatment/Placebo	Relative (95% CI)	Absolute (95% CI)		
**Budesonide Viscous suspension vs Fluticasone Inhalation devices**												
**1**	Randomised trials	Serious[a]	-	Not serious	Not serious	None	56	55	-	MD **0.34 higher.** (0.03 lower to 0.72 higher.)	⨁⨁⨁◯ Moderate	NOT IMPORTANT
**Budesonide Orodispersible tablets vs Placebo**												
**3**	Randomised trials	Serious[a]	Serious[b]	Not serious	Not serious	Publication bias[c]	313	185	-	MD **0.62 lower** (1.04 lower to 0.21 lower)	⨁◯◯◯ Very low	IMPORTANT
**Budesonide Viscous suspension vs Placebo**												
**1**	Randomised trials	Not serious	-	Not serious	Not serious	None	59	29	-	MD **0.55 lower** (1.05 lower to 0.06 lower)	⨁⨁⨁⨁ High	NOT IMPORTANT

CI: Confidence intervals; **MD:** Mean difference. a. Some concerns risk of bias; b. Substantial heterogeneity; c. Publication bias

Table S2F. Quality grading of evidence for treatment modality (combining active ingredient with oesophageal delivery method) on risk for oral candidiasis in patients with eosinophilic esophagitis.

Certainty assessment							№ of patients		Effect		Certainty	Importance
**№ of studies**	**Study design**	**Risk of bias**	**Inconsistency**	**Indirectness**	**Imprecision**	**Other considerations**	**Treatment**	**Treatment/ Placebo**	**Relative (95% CI)**	**Absolute (95% CI)**		
**Fluticasone Inhalation devices vs Placebo**												
**3**	Randomised trials	Serious[a]	Not serious	Not serious	Serious[b]	None	70	50	-	SMD **1.38 SD higher.** (0.29 higher. to 6.57 higher.)	⊕⊕◯◯ Low	NOT IMPORTANT
**Budesonide Inhalation devices vs Placebo**												
**1**	Randomised trials	Serious[a]	-	Not serious	Not serious	None	18	18	-	SMD **1 SD higher.** (0.07 higher. to 14.57 higher.)	⊕⊕⊕◯ Moderate	NOT IMPORTANT
**Budesonide Viscous suspension vs vs Placebo**												
**8**	Randomised trials	Serious[a]	Not serious	Not serious	Not serious	Publication bias[C]	487	257	-	SMD **0.98 SD higher.** (0.04 higher. to 1.92 higher.)	⊕⊕◯◯ Low	IMPORTANT
**Budesonide Viscous suspension vs vs Fluticasone Inhalation devices**												
**1**	Randomised trials	Serious[a]	-	Not serious	Serious[b]	None	56	55	-	SMD **0.52 SD higher.** (0.05 higher. to 5.48 higher.)	⊕⊕◯◯ Low	NOT IMPORTANT
**Budesonide Viscous suspension vs vs Budesonide Inhalation devices**												
**1**	Randomised trials	Serious[a]	-	Not serious	Not serious	None	13	12	-	SMD **0.99 SD higher.** (0.6 higher. to 1.61 higher.)	⊕⊕⊕◯ Moderate	NOT IMPORTANT
**Fluticasone Orodispersible tablets vs Placebo**												
**6**	Randomised trials	Serious[a]	Not serious	Not serious	Serious[b]	Publication bias strongly suspected[c]	98	93	-	SMD **0.82 SD higher.** (0.29 higher. to 2.31 higher.)	⊕◯◯◯ Very low	NOT IMPORTANT
**Budesonide Viscous suspension vs Lansoprazole**												
**1**	Randomised trials	Serious[a]	-	Not serious	Serious[b]	None	15	9	-	SMD **1.6 SD higher.** (0.11 higher. to 23.04 higher.)	⊕⊕◯◯ Low	IMPORTANT
**Budesonide Orodispersible tablets vs Placebo**												
**3**	Randomised trials	Serious[a]	Not serious	Not serious	Serious[b]	None	97	67	-	SMD **1.25 SD higher.** (0.26 higher. to 5.95 higher.)	⊕⊕◯◯ Low	CRITICAL
**Mometasone Inhalation devices vs Placebo**												
**1**	Randomised trials	Serious[a]	-	Not serious	Not serious	None	17	19	-	SMD **0.9 SD higher.** (0.06 higher. to 13.43 higher.)	⊕⊕⊕◯ Moderate	NOT IMPORTANT
**Fluticasone Inhalation devices vs Esomeprazole**												
**1**	Randomised trials	Serious[a]	-	Not serious	Not serious	None	21	21	-	SMD **1 SD higher.** (0.07 higher. to 14.64 higher.)	⊕⊕⊕◯ Moderate	NOT IMPORTANT
**Fluticasone Inhalation devices vs Oral Prednisone**												
**1**	Randomised trials	Serious[a]	Not serious	-	Not serious	None	40	40	-	SMD **1 SD higher.** (0.07 higher. to 14.86 higher.)	⊕⊕⊕◯ Moderate	

CI: Confidence intervals; **SMD:** Standardized mean difference. a. Some concerns risk of bias; b. The direction of the effect is controversial; c. Publication bias

Table S2G. Quality grading of evidence for treatment modality (combining active ingredient with oesophageal delivery method) on risk for oesophageal candidiasis in patients with eosinophilic esophagitis.

Certainty assessment							№ of patients		Effect		Certainty	Importance
**№ of studies**	**Study design**	**Risk of bias**	**Inconsistency**	**Indirectness**	**Imprecision**	**Other considerations**	**Treatment**	**Treatment/Placebo**	**Relative (95% CI)**	**Absolute (95% CI)**		
**Fluticasone Inhalation devices vs Placebo**												
**3**	Randomised trials	Serious[a]	Not serious	Not serious	Serious[b]	Publication bias[C]	70	50	-	SMD **0.67 SD higher.** (0.17 higher. to 2.74 higher.)	⊕◯◯◯ Very low	NOT IMPORTANT
**Budesonide Inhalation devices vs Placebo**												
**1**	Randomised trials	Serious[a]	-	Not serious	Not serious	None	18	18	-	SMD **1 SD higher.** (0.07 higher. to 14.57 higher.)	⊕⊕⊕◯ Moderate	NOT IMPORTANT
**Budesonide Viscous suspension vs Placebo**												
**8**	Randomised trials	Serious[a]	Not serious	Not serious	Not serious	Publication bias[C]	487	257	-	SMD **1.95 SD higher.** (0.00 higher. to 3.93 higher.)	⊕⊕◯◯ Low	IMPORTANT
**Budesonide Viscous suspension vs Fluticasone Inhalation devices**												
**1**	Randomised trials	Serious[a]	-	Not serious	Serious[b]	None	56	55	-	SMD **1.23 SD higher.** (0.52 higher. to 2.91 higher.)	⊕⊕◯◯ Low	NOT IMPORTANT
**Budesonide Viscous suspension vs Budesonide Inhalation devices**												
**1**	Randomised trials	Serious[a]	-	Not serious	Not serious	None	13	12	-	SMD **0.93 SD higher.** (0.06 higher. to 13.88 higher.)	⊕⊕⊕◯ Moderate	NOT IMPORTANT
**Fluticasone Orodispersible tablets vs Placebo**												
**6**	Randomised trials	Serious[a]	Not serious	Not serious	Serious[b]	Publication bias[C]	98	93	-	SMD **0.64 SD higher.** (0.23 higher. to 1.73 higher.)	⊕◯◯◯ Very low	NOT IMPORTANT
**Budesonide Viscous suspension vs Lansoprazole**												
**1**	Randomised trials	Serious[a]	-	Not serious	Not serious	None	15	9	-	SMD **1.6 SD higher.** (0.11 higher. to 23.04 higher.)	⊕⊕⊕◯ Moderate	NOT IMPORTANT
**Budesonide Orodispersible tablets vs Placebo**												
**3**	Randomised trials	Serious[a]	Serious[d]	Not serious	Serious[b]	None	97	67	-	SMD **1.74 SD higher.** (0.17 higher. to 17.41 higher.)	⊕◯◯◯ Very low	CRITICAL
**Mometasone Inhalation devices vs Placebo**												
**1**	Randomised trials	Serious[a]	-	Not serious	Not serious	None	17	19	-	SMD **0.9 SD higher.** (0.06 higher. to 13.43 higher.)	⊕⊕⊕◯ Moderate	NOT IMPORTANT
**Fluticasone Inhalation devices vs Esomeprazole**												
**1**	Randomised trials	Serious[a]	Not -	Not serious	Not serious	None	21	21	-	SMD **0.2 SD higher.** (0.03 higher. to 1.45 higher.)	⊕⊕⊕◯ Moderate	NOT IMPORTANT
**Fluticasone Inhalation devices vs Oral Prednisone**												
**1**	Randomised trials	Serious[a]	-	Not serious	Serious[b]	None	40	40	-	SMD **3.73 SD higher.** (0.43 higher. to 32.17 higher.)	⊕⊕◯◯ Low	NOT IMPORTANT

CI: Confidence intervals; SMD: Standardized mean difference. a. Some concerns risk of bias; b. The direction of the effect is controversial; c. Publication bias; d. Substantial heterogeneity

Table S2H. Quality grading of evidence for treatment modality (combining active ingredient with oesophageal delivery method) on risk for adrenal suppression in patients with eosinophilic esophagitis.

Certainty assessment							№ of patients		Effect		Certainty	Importance
**№ of studies**	**Study design**	**Risk of bias**	**Inconsistency**	**Indirectness**	**Imprecision**	**Other considerations**	**Treatment**	**Treatment/Placebo**	**Relative (95% CI)**	**Absolute (95% CI)**		
**Fluticasone Orodispersible tablets vs. Placebo**												
5	Randomised trials	Not serious	Not serious	Not serious	Not serious	None	79	73	-	SMD **1.06 SD higher.** (0.35 higher. to 3.16 higher.)	⨁⨁⨁⨁ High	NOT IMPORTANT
**Budesonida Orodispersible tablets vs. Placebo**												
3	Randomised trials	Not serious	Not serious	Not serious	Serious[a]	None	97	67	-	SMD **1.25 SD higher.** (0.26 higher. to 5.95 higher.)	⨁⨁⨁◯ Moderate	CRITICAL
**Budesonide Viscous suspension vs. Placebo**												
7	Randomised trials	Serious[b]	Not serious	Not serious	Not serious	None	442	220	-	SMD **0.79 SD higher.** (0.00 higher. to 1.68 higher.)	⨁⨁⨁◯ Moderate	CRITICAL
**Budesonide Viscous suspension vs. Lansoprazole**												
1	Randomised trials	Serious[b]	-	Not serious	Not serious	None	15	9	-	SMD **1.6 SD higher.** (0.11 higher. to 23.04 higher.)	⨁⨁⨁◯ Moderate	NOT IMPORTANT
**Fluticasone Inhalation devices vs. Placebo**												
2	Randomised trials	Serious	Not serious	Not serious	Serious[a]	None	49	35	-	SMD **0.59 SD higher.** (0.12 higher. to 2.99 higher.)	⨁⨁◯◯ Low	NOT IMPORTANT
**Budesonide Viscous suspension vs. Budesonide Inhalation devices**												
1	Randomised trials	Serious[b]	-	Not serious	Serious[a]	None	12	13	-	SMD **0.93 SD higher.** (0.06 higher. to 13.88 higher.)	⨁⨁◯◯ Low	NOT IMPORTANT
**Mometasone Inhalation devices vs. Placebo**												
1	Randomised trials	Serious[b]	-	Not serious	Serious[a]	None	17	19	-	SMD **0.9 SD higher.** (0.06 higher. to 13.43 higher.)	⨁⨁◯◯ Low	NOT IMPORTANT

CI: Confidence intervals; **SMD:** Standardized mean difference. a. The direction of the effect is controversial; b. Publication bias; c. Some concerns risk of bias

Table S3. Comparative efficacy of different topical corticosteroids with low-bioavailability, oral prednisone, proton pump inhibitors and placebo to induce histological remission in patients with active eosinophilic esophagitis. Results are expressed as likelihood of failure in inducing different cut-off point criteria for histological remission, as risk ratio (RR) with their 95% confidence intervals in league tables. Upper right triangle gives the pooled RR from pairwise comparisons (column intervention relative to row), lower left triangle pooled RR from the network meta-analysis (row intervention relative to column). Values below 1 indicate a protective effect against the persistence of histological activity when the comparator intervention was used. Data in bold indicate statistically significant effects.

**<15 to 20 eosinophils per high-power field**					
Placebo	**0.25 (0.12, 0.39)**	**0.35 (0.23, 0.52)**	NA	NA	NA
**0.03 (0.01, 0.09)**	Budesonide	0.70 (0.34, 1.44)	NA	NA	**0.07 (0.01, 0.50)**
**0.03 (0.01, 0.11)**	1.13 (0.23, 5.61)	Fluticasone	1.78 (0.35, 9.06)	1.29 (0.92, 1.81)	NA
**0.02 (<0.01, 0.76)**	0.60 (0.01, 31.07)	0.53 (0.01, 19.59)	Oral Prednisone	NA	NA
**0.01 (<0.01, 0.19)**	0.45 (0.02, 8.35)	2.52 (0.22, 29.06)	1.34 (0.02, 104.61)	Esomeprazole	NA
3.13 (0.04, 266.94)	**112.09 (1.56, 8068.38)**	**99.39 (1.03, 9568.10)**	0.01 (<0.01, 1.80)	**250.22 (1.41, 44498.21)**	Lansoprazole
**<5 to 6 eosinophils per high-power field**					
Placebo	**0.34 (0.20, 0.48)**	**0.39 (0.23, 0.67)**	NA	NA	NA
**0.02 (0.01, 0.08)**	Budesonide	0.83 (0.51, 1.36)	NA	NA	**0.13 (0.04, 0.45)**
**0.03 (0.01, 0.11)**	1.04 (0.17, 6.19)	Fluticasone	1.42 (0.52, 3.89)	1.05 (0.84, 1.32)	NA
**0.02 (<0.01, 0.83)**	0.68 (0.01, 38.50)	0.65 (0.02, 24.45)	Oral Prednisone	NA	NA
**0.02 (<0.01, 0.37)**	0.70 (0.03, 17.90)	1.48 (0.10, 22.05)	0.96 (0.01, 88.53)	Esomeprazole	NA
2.48 (0.02, 300.77)	102.71 (0.99, 10658.34)	98.72 (0.68, 14259.61)	0.01 (<0.01, 3.10)	146.03 (0.51, 41895.51)	Lansoprazole
**<1 eosinophils per high-power field**					
Placebo	**0.61 (0.44, 0.78)**	**0.47 (0.39, 0.57)**	NA	NA	NA
**0.07 (0.02, 0.31)**	Budesonide	NA	NA	NA	**0.67 (0.47, 0.95)**
**0.03 (0.01, 0.14)**	0.46 (0.06, 3.74)	Fluticasone	0.44 (0.18, 1.08)	NA	NA
**0.01 (<0.01, 0.35)**	0.11 (<0.01, 6.76)	0.23 (0.01, 7.23)	Oral Prednisone	NA	NA
NA	NA	NA	NA	Esomeprazole	NA
0.69 (0.01, 82.59)	9.96 (0.11, 937.17)	21.73 (0.15, 3244.37)	0.01 (<0.01, 5.03)	NA	Lansoprazole

Table S4. Comparative efficacy of different modalities to administer topical corticosteroids or placebo to induce histological remission of active eosinophilic esophagitis. Results are expressed as likelihood of failure to induce different cut-off point criteria to define histological remission, as risk ratio (RR) with their 95% confidence intervals in league tables. Upper right triangle gives the pooled RR from pairwise comparisons (column intervention relative to row), lower left triangle pooled RR from the network meta-analysis (row intervention relative to column). Values below 1 indicate a protective effect against the persistence of histological activity when the comparator intervention was used. Data in bold indicate statistically significant effects.

**<15 to 20 eosinophils per high-power field**			
Placebo	**0.17 (0.10, 0.30)**	**0.35 (0.04, 0.65)**	**0.44 (0.23, 0.82)**
**0.02 (0.01, 0.08)**	Orodispersible tablets	NA	NA
**0.03 (0.01, 0.11)**	1.18 (0.22, 6.22)	Viscous suspension	0.63 (0.36, 1.11)
**0.05 (0.01, 0.19)**	2.15 (0.51, 9.13)	1.83 (0.37, 9.10)	Inhalation devices
**<5 to 6 eosinophils per high-power field**			
Placebo	**0.16 (0.06, 0.44)**	**0.47 (0.22, 0.73)**	**0.44 (0.32, 0.60)**
**0.02 (<0.01, 0.07)**	Orodispersible tablets	NA	NA
**0.03 (0.01, 0.10)**	1.69 (0.30, 9.57)	Viscous suspension	0.48 (0.20, 1.16)
**0.04 (0.01, 0.16)**	2.38 (0.50, 11.26)	1.41 (0.28, 7.09)	Inhalation devices
**<1 eosinophil per high-power field**			
Placebo	**0.32 (0.20, 0.53)**	**0.67 (0.42, 0.92)**	**0.50 (0.39, 0.64)**
**0.02 (<0.01, 0.08)**	Orodispersible tablets	NA	**2.84 (1.18, 6.86)**
**0.09 (0.02, 0.33)**	4.50 (0.66, 30.82)	Viscous suspension	0.78 (0.50, 1.22)
**0.08 (0.01, 0.47)**	4.18 (0.57, 30.88)	0.93 (0.13, 6.72)	Inhalation devices

Table S5. Comparative efficacy of different topical corticosteroids with low-bioavailability, oral prednisone, proton pump inhibitors and placebo to induce clinical improvement in active eosinophilic oesophagitis, expressed as standardized mean differences with their 95% confidence intervals in league tables. Upper right triangle gives the pooled standardized mean differences from pairwise comparisons (column intervention relative to row), lower left triangle pooled standardized mean differences from the network meta-analysis (row intervention relative to column).

Placebo	-0.25 (-0.63, 0.12)	NA	-0.57 (-1.24, 0.10)	NA
-0.29 (-0.77, 0.20)	Budesonide	-0.22 (-0.59, 0.16)	NA	NA
0.00 (-1.20, 1.11)	0.29 (-0.70, 1.28)	Fluticasone propionate	NA	0.15 (-0.71, 1.01)
-0.74 (-2.00, 0.52)	-0.45 (-1.81, 0.90)	-0.74 (-2.42, 0.93)	Mometasone furoate	NA
0.31 (-1.11, 1.73)	0.59 (-0.74, 1.93)	-0.30 (-1.20, 0.59)	-1.05 (-2.95, 0.85)	Esomeprazole

Table S6. Comparative efficacy of different modalities to administer topical corticosteroids or placebo to induce clinical improvement in active eosinophilic esophagitis, expressed as standardized mean differences with their 95% confidence intervals in league tables. Upper right triangle gives the pooled standardized mean differences from pairwise comparisons (column intervention relative to row), lower left triangle pooled standardized mean differences from the network meta-analysis (row intervention relative to column). Data in bold indicate statistically significant effects.

Placebo	-0.41<br>(-0.86, 0.04)	-0.02<br>(-0.41, 0.36)	**-0.86**<br>**(-1.46, -0.26)**
-0.26<br>(-1.64, 1.12)	Orodispersible tablets	NA	NA
-0.16<br>(-0.84, 0.52)	0.10<br>(-1.44, 1.64)	Viscous suspension	0.13<br>(-0.70, 0.96)
**-0.91**<br>**(-1.77, -0.06)**	-0.65<br>(-2.28, 0.97)	-0.76<br>(-1.60, 0.09)	Inhalation devices

Table S7. Comparative efficacy of different active ingredients of topical corticosteroids versus comparators for changes induced by therapy in endoscopic features of eosinophilic esophagitis measured by the EREFS system as mean differences with their 95% confidence intervals in league tables. Upper right triangle gives the pooled mean differences from pairwise comparisons (column intervention relative to row), lower left triangle pooled mean differences from the network meta-analysis (row intervention relative to column). Data in bold indicate statistically significant effects.

Placebo	**-0.77 (-1.20, -0.33)**	NA
**-2.82 (-4.18, -1.46)**	Budesonide	0.34 (-0.04, 0.71)
-2.22 (-4.93, 0.48)	0.60 (-1.74, 2.94)	Fluticasone propionate

Table S8. Comparative efficacy of different modalities to administer topical corticosteroids or placebo to induce endoscopic improvement measured by EREFS score in patients with active eosinophilic esophagitis, system as mean differences with their 95% confidence intervals in league tables. Upper right triangle gives the pooled mean differences from pairwise comparisons (column intervention relative to row), lower left triangle pooled mean differences from the network meta-analysis (row intervention relative to column). Data in bold indicate statistically significant effects.

Placebo	**-1.22 (-1.70, -0.74)**	**-0.62 (-1.04, -0.21)**	NA
-2.50 (-5.56, 0.56)	Orodispersible tablets	NA	NA
**-3.09 (-5.10, -1.07)**	-0.59 (-4.25, 3.08)	Viscous suspension	0.34 (-0.04, 0.71)
-2.49 (-6.18, 1.20)	0.01 (-4.78, 4.81)	0.60 (-2.49, 3.69)	Inhalation devices

Table S9. Comparative efficacy of different topical corticosteroids-based presentations versus comparators for changes induced by therapy in endoscopic features of eosinophilic esophagitis measured by the EREFS system as mean differences with their 95% confidence intervals in league tables. Upper right triangle gives the pooled mean differences from pairwise comparisons (column intervention relative to row), lower left triangle pooled mean differences from the network meta-analysis (row intervention relative to column). Data in bold indicate statistically significant effects.

Placebo	-1.22 (-1.70, 0.74)	**-0.62 (-1.04, -0.21)**	NA
-2.50 (-5.56, 0.56)	Budesonide orodispersible tablets	NA	NA
**-3.09 (-5.10, -1.07)**	-0.59 (-4.25, 3.08)	Budesonide viscous suspension	0.34 (-0.04, 0.71)
-2.49 (-6.18, 1.20)	0.01 (-4.78, 4.81)	0.60 (-2.49, 3.69)	Fluticasone inhalation devices

Table S10. Effectiveness ranking of different active ingredients, irrespective of delivery method, proton pump inhibitors and placebo to induce histological remission of active eosinophilic esophagitis, defined by different cut-off criteria of eosinophil density in esophageal biopsies.

**<15 to 20 eosinophils per high-power field**					
	**Rank statistics**			**Probabilities**	
	**Mean**	**Median**	**95%CIs**	**Best**	**SUCRA**
**Esomeprazole**	2.0	3.5	1.0 – 5.0	0.44	0.81
**Prednisone**	2.3	3.0	2.0 – 4.0	0.40	0.73
**Budesonide**	2.8	2.5	1.0 – 6.0	0.12	0.64
**Fluticasone**	3.0	3.5	1.0 – 4.0	0.03	0.61
**Placebo**	5.3	6.0	1.0 – 6.0	0.00	0.15
**Lansoprazole**	5.6	5.0	1.0 – 5.0	0.00	0.08
**<5 to 6 eosinophils per high-power field**					
	**Rank statistics**			**Probabilities**	
	**Mean**	**Median**	**95%CIs**	**Best**	**SUCRA**
**Prednisone**	2.4	3.0	1.0 – 4.0	0.40	0.73
**Esomeprazole**	2.3	3.0	2.0 – 4.0	0.36	0.73
**Fluticasone**	2.7	4.0	1.0 – 5.0	0.08	0.66
**Budesonide**	2.8	2.5	1.0 – 6.0	0.15	0.65
**Placebo**	5.3	6.0	1.0 – 6.0	0.00	0.14
**Lansoprazole**	5.5	5.0	1.0 – 5.0	0.00	0.09
**<1 eosinophil per high-power field**					
	**Rank statistics**			**Probabilities**	
	**Mean**	**Median**	**95%CIs**	**Best**	**SUCRA**
**Prednisone**	1.4	3.0	1.0 – 4.0	0.74	0.89
**Fluticasone**	2.1	2.0	1.0 – 5.0	0.15	0.72
**Budesonide**	2.8	3.0	1.0 – 4.0	0.07	0.56
**Lansoprazole**	4.1	3.0	1.0 – 4.0	0.05	0.23
**Placebo**	4.6	5.0	1.0 – 5.0	0.00	0.11

Table S11. Effectiveness ranking of different methods for esophageal delivery of topical corticosteroids and placebo to induce histological remission of active eosinophilic esophagitis, defined by different cut-off criteria of eosinophil density in esophageal biopsies.

**<15 to 20 eosinophils per high-power field**					
	**Rank statistics**			**Probabilities**	
	**Mean**	**Median**	**95%CIs**	**Best**	**SUCRA**
**Orodispersible tablets**	1.4	2.5	1.0 – 3.0	0.64	0.85
**Viscous suspension**	2.0	2.0	1.0 – 2.0	0.29	0.67
**Inhalation devices**	2.6	2.0	1.0 – 3.0	0.07	0.48
**Placebo**	4.0	4.0	1.0 – 4.0	0.00	0.00
**<5 to 6 eosinophils per high-power field**					
	**Rank statistics**			**Probabilities**	
	**Mean**	**Median**	**95%CIs**	**Best**	**SUCRA**
**Orodispersible tablets**	1.3	2.5	1.0 – 3.0	0.72	0.88
**Viscous suspension**	2.2	2.0	1.0 – 2.0	0.19	0.60
**Inhalation devices**	2.5	2.0	1.0 – 3.0	0.09	0.51
**Placebo**	4.0	4.0	1.0 – 4.0	0.00	0.00
**<1 eosinophil per high-power field**					
	**Rank statistics**			**Probabilities**	
	**Mean**	**Median**	**95%CIs**	**Best**	**SUCRA**
**Orodispersible tablets**	1.1	2.5	– 4.0	0.86	0.95
**Inhalation devices**	2.0	2.0	1.0 – 3.0	0.14	0.68
**Viscous suspension**	2.9	2.5	1.0 – 3.0	0.00	0.37
**Placebo**	4.0	3.5	1.0 – 4.0	0.00	0.00

Table S12. Effectiveness ranking of different presentations of topical corticosteroids versus comparators to induce clinical improvement in active eosinophilic esophagitis.

	Rank statistics			Probabilities	
	Mean	Median	95%CIs	Best	SUCRA
**Budesonide inhalation devices**	1.1	7.0	1.0 – 7.0	0.93	0.99
**Mometosone inhalation devices**	2.5	6.0	1.0 – 6.0	0.07	0.76
**Budesonide orodispersible tablets**	3.6	4.0	1.0 – 5.0	0.00	0.56
**Budesonide viscous suspension**	4.2	4.0	1.0 – 5.0	0.00	0.46
**Fluticasone inhalation devices**	5.5	3.0	1.0 – 5.0	0.00	0.25
**Placebo**	4.7	3.0	1.0 – 7.0	0.00	0.38
**Esomeprazole**	6.3	5.0	1.0 – 6.0	0.00	0.11

Table S13. Effectiveness ranking of different presentations of topical corticosteroids versus comparators to endoscopic features measured by EREFS score in active eosinophilic esophagitis.

	Rank statistics			Probabilities	
	**Mean**	**Median**	**95%CIs**	**Best**	**SUCRA**
**Budesonide viscous suspension**	1.7	2.5	1.0 – 4.0	0.40	0.76
**Budesonide orodispersible tablets**	2.2	2.0	1.0 – 3.0	0.32	0.61
**Fluticasone inhalation devices**	2.2	2.0	2.0 – 3.0	0.28	0.58
**Placebo**	3.9	3.5	1.0 – 4.0	0.00	0.11

Table S14. Comparative risk of different active ingredients of swallowed topical corticosteroids versus comparators in the risk of adverse events resulting from induction of remission therapy in patients with active eosinophilic esophagitis. Results are expressed as risk ratios (RR) with their 95% confidence intervals in league tables. Upper right triangle gives the pooled RR from pairwise comparisons (column intervention relative to row), lower left triangle pooled RR from the network meta-analysis (row intervention relative to column). Values greater than 1 indicate a protective effect against the persistence of histological activity when the comparator intervention was used.

Oral candidiasis						
Placebo	1.04 (0.23, 1.84)	0.96 (0.41, 2.28)	0.90 (0.06, 13.43)	NA	NA	NA
1.56 (0.68, 3.56)	Budesonide	0.52 (0.05, 5.48)	NA	NA	NA	1.60 (0.11, 23.04)
1.47 (0.49, 4.42)	0.94 (0.26, 3.37)	Fluticasone propionate	NA	1.00 (0.07. 14.86)	1.00 (0.07, 14.64)	NA
1.11 (0.02, 59.20)	0.71 (0.01, 41.34)	0.76 (0.01, 46.64)	Mometasone furoate	NA	NA	NA
1.47 (0.02, 88.36)	0.94 (0.01, 59.56)	1.00 (0.02, 51.63)	1.32 (<0.01, 396.98)	Oral prednisone	NA	NA
1.47 (0.02, 90.18)	0.94 (0.01, 60.77)	1.00 (0.02, 52.73)	0.76 (<0.01, 230.50)	1.00 (<0.01, 268.46)	Esomeprazole	NA
0.79 (0.03, 23.90)	0.51 (0.02, 13.84)	0.54 (0.02, 18.56)	1.40 (0.01, 263.01)	1.86 (0.01, 396.98)	0.54 (<0.01, 109.55)	Lansoprazole

Esophageal candidiasis						
Placebo	1.89 (0.44, 3.34)	0.65 (0.29, 1.46)	0.90 (0.06, 13.43)	NA	NA	NA
0.98 (0.37, 2.61)	Budesonide	1.23 (0.52, 2.91)	NA	NA	NA	1.60 (0.11, 23.04)
2.24 (0.73, 6.90)	2.28 (0.63, 8.29)	Fluticasone propionate	NA	3.73 (0.43, 32.17)	0.20 (0.03, 1.45)	NA
0.35 (0.01, 13.76)	0.36 (0.01, 15.92)	0.16 (<0.01, 7.26)	Mometasone furoate	NA	NA	NA
0.22 (0.01, 7.99)	0.23 (0.01, 8.60)	0.10 (<0.01, 2.97)	0.64 (<0.01, 106.36)	Oral prednisone	NA	NA
0.12 (<0.01, 4.40)	0.13 (<0.01, 4.74)	18.03 (0.61, 532.26)	2.83 (0.02, 471.13)	1.81 (0.01, 217.41)	Esomeprazole	NA
1.60 (0.02, 136.05)	1.63 (0.02, 124.29)	0.71 (0.01, 65.65)	0.22 (<0.01, 69.69)	0.14 (<0.01, 39.89)	12.88 (0.05, 3652.84)	Lansoprazole

Adrenal suppression				
Placebo	0.79 (0.06, 1.52)	1.18 (0.45, 3.08)	1.11 (0.07, 17.04)	NA
1.17 (0.41,3.31)	Budesonide	NA	NA	0.63 (0.04, 9.40)
1.17 (0.31,4.40)	1.00 (0.19,5.42)	Fluticasone propionate	NA	NA
1.11 (0.02,59.20)	0.96 (0.02,58.12)	0.95 (0.01,62.70)	Mometasone furoate	NA
1.90 (0.03,119.00)	1.63 (0.03,89.28)	1.63 (0.02,125.04)	0.59 (<0.01,181.33)	Lansoprazole

Table S15. Comparative risk of different esophageal delivery methods for swallowed topical corticosteroids, irrespective of active ingredients versus comparators in the risk of adverse events resulting from induction of remission therapy in patients with active eosinophilic esophagitis. Results are expressed as risk ratios (RR) with their 95% confidence intervals in league tables. Upper right triangle gives the pooled RR from pairwise comparisons (column intervention relative to row), lower left triangle pooled RR from the network meta-analysis (row intervention relative to column). Values greater than 1 indicate a protective effect against the persistence of histological activity when the comparator intervention was used.

**Oral candidiasis**			
Placebo	1.00 (0.34, 2.93)	0.98 (0.04, 1.92)	0.93 (0.39, 2.21)
0.95 (0.25, 3.58)	Inhalation devices	0.93 (0.06, 13.88)	1.00 (0.07, 14.86)
1.63 (0.66, 4.03)	1.72 (0.42, 6.96)	Viscous suspension	NA
1.68 (0.52, 5.39)	1.77 (0.33, 9.49)	1.03 (0.24, 4.43)	Orodispersible tablets

**Esophageal candidiasis**			
Placebo	0.76 (0.25, 2.35)	1.95 (0.00, 3.93)	0.98 (0.40, 2.38)
1.72 (0.47, 6.33)	Inhalation devices	1.20 (0.53, 2.73)	3.73 (0.43, 32.17)
1.18 (0.36, 3.88)	0.69 (0.18, 2.68)	Viscous suspension	NA
1.18 (0.35, 4.02)	0.68 (0.13, 3.64)	1.00 (0.19, 5.28)	Orodispersible tablets

**Adrenal suppression**			
Placebo	0.88 (0.34, 2.26)	0.79 (0.00, 1.68)	0.63 (0.04, 9.40)
0.90 (0.25, 3.22)	Orodispersible tablets	NA	NA
1.19 (0.37, 3.78)	1.32 (0.24, 7.38)	Viscous suspension	1.08 (0.07, 16.07)
2.14 (0.34, 13.35)	2.37 (0.25, 22.11)	1.80 (0.24, 13.78)	Inhalation devices

Table S16. Heterogeneity statistics for each comparison for different: A. active ingredient; B. esophageal drug delivery modality; and C. combination of both criteria on histological remission, defined by several cut-off points.

**A. Active ingredient**				
**<15 to 20 eosinophils per high power field**				
	**Q (df)**	**I^2**	**τ2**	**p**
**Fluticasone - Placebo**	19.18 (8)	58.30%	0.20	0.014
**Budesonide - Placebo**	80.40 (8)	91.30%	0.58	0.000
**Budesonide - Lanzoprazole**	0.00 (0)	-	-	0.000
**Budesonide - Fluticasone**	0.00 (0)	-	-	0.000
**Fluticasone - Esomeprazole**	0.74 (1)	0.00%	0.00	0.388
**Prednisone - Fluticasone**	0.00 (0)	-	-	0.000
**<5 to 6 eosinophils per high power field**				
	**Q (df)**	**I^2**	**τ2**	**p**
**Fluticasone - Placebo**	37.88 (6)	84.20%	0.38	0.000
**Budesonide - Placebo**	104.05 (11)	90.40%	0.40	0.000
**Budesonide - Lansoprazole**	0.00 (0)	-	-	0.000
**Budesonide - Fluticasone**	0.00 (0)	-	-	0.000
**Fluticasone - Esomeprazole**	0.07 (1)	0.00%	0.00	0.793
**Prednisone- Fluticasone**	0.00 (0)	-	-	0.000
**<1 eosinophil per high power field**				
	**Q (df)**	**I^2**	**τ2**	**p**
**Fluticasone-Placebo**	9.54 (8)	16.20%	0.01	0.299
**Budesonide-Placebo**	58.63 (7)	89.80%	0.12	0.000
**Budesonide-Lansoprazole**	0.00 (0)	-	-	0.000
**Fluticasone-Prednisone**	0.00 (0)	-	-	0.000

**B. Modality to administer the drug**				
**<15 to 20 eosinophils per high power field**				
	**Q (df)**	**I^2**	**τ2**	**p**
**Inhalation devices - Placebo**	10.43 (3)	71.20%	0.27	0.000
**Viscous suspension - Placebo**	50.27 (4)	94.00%	0.41	0.000
**Orodispersible tablets -Placebo**	17.40 (8)	54.00%	0.33	0.026
**Inhalation devices – Viscous suspension**	0.19 (1)	0.00%	0.00	0.660
**<5 to 6 eosinophils per high power field**				
	**Q (df)**	**I^2**	**τ2**	**p**
**Inhalation devices - Placebo**	3.06 (3)	1.80%	0.00	0.383
**Viscous suspension - Placebo**	55.09 (8)	87.30%	0.21	0.000
**Orodispersible tablets - Placebo**	73.91 (6)	91.90%	1.65	0.000
**Inhalation devices - Viscous suspension**	0.00 (0)	-	-	0.000
**<1 eosinophil per high power field**				
	**Q (df)**	**I^2**	**τ2**	**p**
**Inhalation devices - Placebo**	0.47 (2)	0.00%	0.00	0.790
**Viscous suspension - Placebo**	28.47 (5)	10.01%	0.01	0.351
**Orodispersible tablets - Placebo**	23.75 (6)	85.90%	0.07	0.000
**Inhalation devices - Viscous suspension**	1.60 (1)	37.70%	0.05	0.205
**Inhalation devices - Orodispersible tablets**	0.00 (0)	-	-	0.000

C. Combination of both criteria (active ingredient & oesophageal delivery method)				
**<15 to 20 eosinophils per high power field**				
	**Q (df)**	**I^2**	**τ2**	**p**
**Fluticasone inhalation devices - Placebo**	6.82 (2)	70.70%	0.19	0.033
**Budesonide inhalation devices - Budesonide viscous suspension**	0.00 (0)	-	-	0.000
**Budesonide viscous suspension - Placebo**	50.27 (4)	94.00%	0.41	0.000
**Fluticasone orodispersible tablets -Placebo**	5.73 (5)	12.70%	0.03	0.333
**Budesonide viscous suspension - Lansoprazole**	0.00 (0)	-	-	0.000
**Budesonide orodispersible tablets -Placebo**	0.22 (2)	0.00%	0.00	0.896
**Budesonide viscous suspension -Fluticasone inhalation devices**	0.00 (0)	-	-	0.000
**Fluticasone inhalation devices -Esomeprazole**	0.74(1)	0.00%	0.00	0.388
**Prednisone orodispersible tablets -Fluticasone inhalation devices**	0.00 (0)	-	-	0.000
**Budesonide inhalation devices - Placebo**	0.00 (0)	-	-	0.000
**<5 to 6 eosinophils per high power field**				
	**Q (df)**	**I^2**	**τ2**	**p**
**Fluticasone inhalation devices - Placebo**	2.07 (2)	3.20%	0.00	0.356
**Budesonide inhalation devices - Budesonide viscous suspension**	0.00 (0)	-	-	0.000
**Budesonide viscous suspension - Placebo**	55.09 (8)	87.30%	0.21	0.000
**Fluticasone orodispersible tablets -Placebo**	29.66 (3)	89.90%	0.95	0.000
**Budesonide viscous suspension - Lansoprazole**	0.00 (0)	-	-	0.000
**Budesonide orodispersible tablets -Placebo**	0.19 (2)	0.00%	0.00	0.911
**Fluticasone inhalation devices -Esomeprazole**	0.07 (1)	0.00%	0.00	0.793
**Prednisone orodispersible tablets -Fluticasone inhalation devices**	0.00 (0)	-	-	0.000
**Budesonide inhalation devices - Placebo**	0.00 (0)	-	-	0.000
**<1 eosinophil per high power field**				
	**Q (df)**	**I^2**	**τ2**	**p**
**Fluticasone inhalation devices - Placebo**	0.47 (2)	0.00%	0.00	0.790
**Budesonide inhalation devices - Budesonide viscous suspension**	0.00 (0)	-	-	0.000
**Budesonide viscous suspension - Placebo**	28.47 (5)	85.90%	0.07	0.000
**Fluticasone orodispersible tablets –Placebo**	8.88 (5)	43.70%	0.08	0.114
**Budesonide viscous suspension – Lansoprazole**	0.00 (0)	-	-	0.000

Table S17. Heterogeneity statistics for each comparison for different: A. active ingredient; B. esophageal drug delivery modality, and C. combination of both criteria on clinical improvement.

**A. Active ingredient**				
	**Q (df)**	**I^2**	**τ2**	**p**
**Fluticasone - Esomeprazole**	3.32 (1)	69.90%	0.27	0.069
**Budesonide - Fluticasone**	0.00 (0)	-	-	<0.001
**Budesonide - Placebo**	17.36 (4)	77.00%	0.13	0.002
**Mometasone - Placebo**	0.00 (0)	-	-	<0.001

**B. Modality to administer the drug**				
	**Q (df)**	**I^2**	**τ2**	**P**
**Inhalation devices - Placebo**	1.51 (1)	33.80%	0.06	0.219
**Viscous suspension - Placebo**	7.60 (2)	73.70%	0.08	0.022
**Orodispersible tablets - Placebo**	0.00 (0)	-	-	<0.001
**Inhalation devices - Viscous suspension**	3.60 (1)	72.20%	0.27	0.058

**C. Combination of both criteria (active ingredient/modality to administer the drug)**				
	**Q (df)**	**I^2**	**τ2**	**p**
**Fluticasone Inhalation devices -Esomeprazole**	3.32 (1)	69.90%	0.27	0.069
**Budesonide Inhalation devices - Budesonide viscous suspension**	0.00 (0)	-	-	<0.001
**Budesonide viscous suspension - Placebo**	7.60 (2)	73.70%	0.08	0.022
**Fluticasone inhalation devices -Budesonide viscous suspension**	0.00 (0)	-	-	<0.001
**Budesonide inhalation devices - Placebo**	0.00 (0)	-	-	<0.001
**Budesonide orodispersible tablets -Placebo**	0.00 (0)	-	-	<0.001
**Mometasone inhalation devices - Placebo**	0.00 (0)	-	-	<0.001
